# Cell Membrane Fragment-Wrapped Parenteral Nanoemulsions: A New Drug Delivery Tool to Target Gliomas

**DOI:** 10.3390/cells13070641

**Published:** 2024-04-06

**Authors:** Chiara Dianzani, Annalisa Bozza, Valentina Bordano, Luigi Cangemi, Chiara Ferraris, Federica Foglietta, Chiara Monge, Margherita Gallicchio, Stefania Pizzimenti, Elisabetta Marini, Elisabetta Muntoni, Maria Carmen Valsania, Luigi Battaglia

**Affiliations:** 1 Department of Drug Science and Technology, University of Turin, via Pietro Giuria 9, 10124 Turin, Italy; chiara.dianzani@unito.it (C.D.); annalisa.bozza@unito.it (A.B.); valentina.bordano@unito.it (V.B.); luigi.cangemi@unito.it (L.C.); chiara.viola.isabella@gmail.com (C.F.); federica.foglietta@unito.it (F.F.); chiara.monge@unito.it (C.M.); margherita.gallicchio@unito.it (M.G.); elisabetta.marini@unito.it (E.M.); elisabetta.muntoni@unito.it (E.M.); 2 Department of Clinical and Biological Sciences, University of Turin, Corso Raffaello 30, 10124 Turin, Italy; stefania.pizzimenti@unito.it; 3 Department of Chemistry, University of Turin, Via Quarello 15/a, 10135 Turin, Italy; mariacarmen.valsania@unito.it; 4 Nanostructured Interfaces and Surfaces (NIS) Interdepartmental Centre, University of Turin, 10124 Turin, Italy

**Keywords:** nanoemulsions, glioma, cell membrane fragments

## Abstract

Poor prognosis in high-grade gliomas is mainly due to fatal relapse after surgical resection in the absence of efficient chemotherapy, which is severely hampered by the blood–brain barrier. However, the leaky blood–brain–tumour barrier forms upon tumour growth and vascularization, allowing targeted nanocarrier-mediated drug delivery. The homotypic targeting ability of cell-membrane fragments obtained from cancer cells means that these fragments can be exploited to this aim. In this experimental work, injectable nanoemulsions, which have a long history of safe clinic usage, have been wrapped in glioma-cell membrane fragments via co-extrusion to give targeted, homogeneously sized, sterile formulations. These systems were then loaded with three different chemotherapeutics, in the form of hydrophobic ion pairs that can be released into the target site thanks to interactions with physiological components. The numerous assays performed in two-dimensional (2D) and three-dimensional (3D) cell models demonstrate that the proposed approach is a versatile drug-delivery platform with chemo-tactic properties towards glioma cells, with adhesive interactions between the target cell and the cell membrane fragments most likely being responsible for the effect. This approach’s promising translational perspectives towards personalized nanomedicine mean that further *in vivo* studies are foreseen for the future.

## 1. Introduction

Gliomas are the most frequent brain tumours, which originate from glial cells, and range from low to high grade. Surgery is the first-line treatment and several techniques have been designed to refine tumour resection, with a positive prognostic effect: neuronavigation, use of 5-aminolevulinic acid, and intra-operative magnetic resonance imaging. Nonetheless, relapses after tumour-mass surgical removal are associated with a negative prognosis in high-grade gliomas. To this aim, combined chemotherapy/radiotherapy approaches are currently practiced after surgery as adjuvant, to target residual tumour cells. However, radiotherapy is related to important side effects, such as post-radiation leuko-encephalopathy, nerve damage, hair loss, vomiting, infertility, and skin rash. Chemotherapy is currently only regarded as palliative care, mainly due to poor tumour accumulation and chemo-resistance [1]. Indeed, the blood–brain barrier (BBB), due to the tight junctions among endothelial cells in brain vasculature, hampers brain access to nearly 98% of potential chemotherapeutic agents. Accordingly, despite the several drug candidates undergoing clinical trials, only three chemotherapeutics are currently approved by the Food and Drug Administration (FDA); oral temozolomide, IV bevacizumab (Avastin^®^), and carmustine-loaded biodegradable wafers (Gliadel^®^), which are implanted into the brain after surgery [2].

Nanomedicine has already been proposed for the treatment of brain pathologies, thanks to its passive- and active-targeting capabilities [3]. Nonetheless, the poor translational potential of most preclinical nanotechnologies to date has meant that only liposomal doxorubicin (Caelyx^®^) [4,5] and irinotecan (Onivyde^®^) [6] have entered clinical trials for the off-label chemotherapy of gliomas. On the other hand, injectable lipid nanoemulsions, which have a long history of safe clinical usage in parenteral nutrition, have recently been proposed for drug delivery, with several formulations reaching the market as vehicles for anaesthetics, hypnotics, corticosteroids, and non-steroidal anti-inflammatory drugs (NSAIDs) [7,8]. In this context, cell-membrane fragments (CMFs), obtained from various source cells (e.g., red blood cells, immune cells, cancer cells, platelets, and fusion-cell membranes), can be endowed with important properties, such as long blood circulation, immune escape and targeting abilities [9,10,11,12,13,14,15,16], and have recently been used to wrap injectable nanoemulsions via simple co-extrusion with syringe filters (220 nm) [17].

Therefore, this experimental work proposes CMF-wrapped nanoemulsions as a versatile platform for the delivery of traditional chemotherapeutic drugs (doxorubicin, irinotecan, cisplatin) against gliomas by exploiting homotypic targeting, which works via interactions between the adhesion proteins that are abundant on cancer cell membranes. A reproducible methodology, with translational potential, has been optimized and presented in this work. To this aim, the aforementioned water-soluble drugs were loaded into the nanoemulsion oil phase via the formation of hydrophobic ion pairs (HIPs) with negatively charged surfactants. This process occurs without the chemical modification of the compounds, which can be released into the physiological environment after HIP displacement. In view of future studies in F98/Fischer rats, which are an orthotopic immuno-competent syngeneic glioma model [18,19] that truly recapitulates the human condition, F98 glioma cells were selected as the CMF source, while CMFs from rat red blood cells (RBCs) were used as models for additional studies. Engineered formulations underwent physico-chemical characterization, and preliminary *in vitro* studies on available F98 cell models were performed.

## 2. Materials and Methods

### 2.1. Chemicals

3-(4,5-dimethyl thiazol-2-yl)-2,5-diphenyltetrazolium bromide (MTT), 6-coumarin (6-CUM), acetonitrile, chloroform, crystal violet, dimethylsulfoxide (DMSO), ethanol, fluorescein isothiocyanate (FITC), FITC-dextran 500,000 MW, methanol, potassium nitrate (KNO_3_), and trypsin were obtained from Sigma-Aldrich (St. Louis, MO, USA). Diethyldithiocarbamate (DDTC) was obtained from Fluka (Buchs, Switzerland), and 25% ammonia, formic acid, hydrochloric acid (HCl), the Millipore MilliQ system (for deionized water), phosphotungstic acid, sodium dioctylsulfosuccinate (AOT), and trometamol (TRIS) were obtained from Merck (Darmstadt, Germany). Ethylenediaminetetraacetic acid (EDTA), hexa-hydrate magnesium chloride (MgCl_2_), potassium chloride (KCl), sodium chloride (NaCl), and sodium hydroxide (NaOH) were obtained from Carlo Erba (Cornaredo, Italy). 2-heptanesulfonic acid sodium salt, 4′,6-Diamidino-2-phenylindole (DAPI), 7-ethyl-10-hydroxycamptothecin (SN-38), cisplatin, irinotecan hydrochloride, and silver hexafluorophosphate (AgPF_6_) were obtained from VWR International (Radnor, PA, USA). Isopropanol and trifluoroacetic acid (TFA) were obtained from Alfa Aesar (Haverhill, MA, USA). Intralipid^®^ 10% (IL) was obtained from BBraun, and 220 nm sterile filters were obtained from GVS (Bologna, Italy). The CD324 (E-Cadherin) monoclonal antibody (DECMA)—Alexa Fluor™ 488 was obtained from eBioscience, Thermo Fisher Scientific (Milano, Italy). CD47/MER6 polyclonal antibody, FITC conjugated, was obtained from CliniSciences, (Guidonia Montecelio, Italy). Doxorubicin hydrochloride was kindly gifted from Farmitalia (Milan, Italy). An FITC annexin V Apoptosis Detection Kit (Cat. No. 556547) was obtained by BD Biosciences (Milan, Italy). All other chemicals were of analytical grade and used without any further purification.

### 2.2. Cells

F98 glioma rat cells were purchased from the American type culture collection (ATCC; Manassas, VA, USA). F98 cells were cultured in Dulbecco’s Modified Eagle Medium (DMEM—Sigma-Aldrich, St. Louis, MO, USA). All culture media were supplemented with 10% foetal calf serum (FCS; PAA Laboratories, Pasching, Austria), penicillin/streptomycin (100 units/mL) and L-glutamine (2 mmol/L) (both from Sigma-Aldrich, St. Louis, MO, USA). Cells were cultured in a 5% CO_2_, 37 °C incubator.

Human cerebral microvascular endothelial cells (hCMEC/D3) were purchased from Millipore Corporation (Temecula, CA, USA). hCMEC/D3 were cultured in EndoGro^TM^ basal medium with 0.2% bovine brain extract, 0.5 ng/mL recombinant epidermal growth factor (rh-EGF), 50 µg/mL ascorbic acid, 10 mM L-glutamine, 1 µg/mL hydrocortisone hemisuccinate, 0.75 IU/mL heparin sulfate, and 5% FCS, at 37 °C and with a humidified atmosphere of 5% CO_2_.

RBCs were isolated from rat blood, which had previously been collected after animal sacrifice in heparinized tubes, according to a protocol approved by the Italian Ministry of Health (56105.N.ZMT approved on 23 June 2018 and 56105.N.WSP approved on 14 July 2023). Purified RBCs were obtained via centrifugation of 3 mL blood at 865× *g* for 3 min (Rotofix 32 centrifuge, Hettich, Tuttingen, Germany), followed by 3 washing cycles with 1.5 mL normal saline.

### 2.3. Methods

#### 2.3.1. Wrapping IL with CMF

CMFs were obtained by working under a sterile hood and in an ice bath. F98 CMFs were obtained from F98 cultured cells; F98 cell pellets (approximately 10^6^ F98 cells) were detached from flasks with sterile 0.58 mg/mL EDTA and washed 3 times with PBS via centrifuging at 500× *g* [17]. RBC CMFs were obtained from purified rat RBCs; RBC pellets were isolated via the centrifugation of approximately 3 mL RBC suspension at 865× *g* for 3 min.

In both cases, cell pellets were re-suspended, using a micropipette, in 1 mL of 0.75 mg/mL KCl and 0.41 mg/mL MgCl_2_ in 0.02M TRIS-HCl sterile hypotonic buffer, and underwent lysis in a glass-homogenizer (20 strokes), followed by centrifugation at 3200× *g* for 5 min (Allegra^®^ 64R centrifuge, Beckman Coulter, Palo Alto, CA, USA), in order to extract CMFs into the supernatant. This procedure was repeated twice, and CMFs were then isolated from the supernatant via centrifugation at 19,800× *g* for 20 min (Allegra^®^ 64R centrifuge, Beckman Coulter, Palo Alto, CA, USA), and the CMF suspension was obtained from the pellet using a micropipette in 1 mL of sterile normal saline. In the case of RBC CMFs, the pellet was washed a further 3 times with normal saline.

F98 CMFs were characterized as is, while, for RBC CMF, 50 μL of CMF suspension was diluted with 1 mL normal saline prior to physico-chemical characterization.

The wrapping of IL with RBC/F98 CMFs was achieved via co-extrusion with the 100 μL RBC CMF or 200 μL F98 CMF suspensions, respectively, and 1 mL IL, using 220 nm sterile filters [17].

#### 2.3.2. FITC Labelling of RBC

In order to track IL wrapping with RBC CMFs, purified rat RBCs were labelled with FITC, according to a partially modified literature method [20]. Briefly, 3 mL of purified rat RBC suspension was mixed with 500 μL of a 2 mg/mL solution of FITC in DMSO, and left to react overnight at 2–8 °C. The reaction mixture was then centrifuged at 865× *g* for 3 min (Rotofix 32 centrifuge, Hettich, Tuttingen, Germany) and the precipitate, made up of FITC-labelled RBCs, was washed three times with 1.5 mL of normal saline.

#### 2.3.3. Drug HIP Preparation and Characterization

To promote drug entrapment within the oily droplets of the nanoemulsions, the selected drugs (doxorubicin, irinotecan, cisplatin) underwent HIP formation with AOT, following literature methods. Briefly, doxorubicin hydrochloride and irinotecan hydrochloride were separately dissolved in distilled water, at 10 and 4 mg/mL, respectively. Their corresponding HIPs were precipitated via the addition of an AOT solution (4.5 mg/mL) in a 1:1 molar ratio, followed by centrifugation at 34,000× *g* for 5 min (Allegra^®^ 64R centrifuge, Beckman Coulter, Palo Alto, CA, USA) [21,22]. In the case of cisplatin, a 2 mg/mL solution in water was added to 10 mg/mL AgPF_6_ (1:2 molar ratio), and left under stirring overnight, in order to precipitate AgCl, which was then removed via centrifugation at 53,000× *g* for 30 min (Allegra^®^ 64R centrifuge, Beckman Coulter, Palo Alto, CA, USA). The supernatant, containing the platinum, was added to a 4.5 mg/mL AOT solution (platinum–AOT molar ratio 1:2), and kept under magnetic stirring for 30 min, which allowed cisplatin–AOT precipitation to occur, prior to centrifugation at 34,000× *g* for 10 min (Allegra^®^ 64R centrifuge, Beckman Coulter, Palo Alto, CA, USA) [23]. The three precipitated HIPs were further washed with distilled water, and then dried under a nitrogen flow.

#### 2.3.4. Loading of Drug HIP into Nanoemulsions

The HIPs of the three drugs were pre-dissolved separately in a small volume of a suitable solvent: 0.5 mg doxorubicin–AOT and 2 mg irinotecan-AOT in 20 μL DMSO (25 and 100 mg/mL, respectively), 7.4 mg cisplatin–AOT in 40 μL ethanol (185 mg/mL). These solutions were slowly incorporated into 1 mL of nanoemulsions via gentle mixing with a micropipette, until the insoluble crystals disappeared.

#### 2.3.5. Fluorescence Labelling of Nanoemulsions

In order to perform uptake studies in F98 glioma cells, uncoated and F98 CMF-wrapped IL were both labelled with a 0.1 mg/mL 6-CUM fluorescent probe [17]. To this aim, 50 μL of a 2 mg/mL stock solution of 6-CUM in DMSO were added to 1 mL of nanoemulsion, followed by mixing with a micropipette.

#### 2.3.6. Characterization of Formulations

##### 2.3.6.1. Particle Size, Zeta Potential, Electronic Microscopy

The dynamic light scattering technique (DLS; 90 Plus, Brookhaven, NY, USA) was used to determine the mean droplet size, polydispersity index (PDI) and Zeta potential of the IL-based formulations at 25 °C, in triplicate. The measurement angles were 90° for particle size and 15° for Zeta potential. Optical microscopy was performed on a DM2500 microscope (Leica Microsystems, Wetzlar, Germany), equipped with a Moticam 480 camera (Motic, Barcelona, Spain). Transmission electron microscopy (TEM, high-resolution JEOL 300 kV) was performed via IL negative staining with 1% phosphotungstic acid [17].

##### 2.3.6.2. Protein Content

The proteins grafted to the IL surface were quantified using high pressure liquid chromatography (HPLC), after nanoemulsion ultracentrifugation and protein extraction [17].

The protein content of CMFs was determined as follows; 100 μL CMFs were separated from the supernatant via centrifugation at 14,300× *g* (MPW55, Medical Instruments, San Lazzaro di Savena, Italy) for 5 min, and the pellet was extracted using an established volume of protein extraction solution, prior to HPLC injection (50 μL in the case of F98, 300 μL in the case of RBC).

The proteins grafted to the IL droplet of CMF-wrapped IL were determined as follows; 1 mL of the formulations obtained was centrifuged at 62,000× *g* (Allegra^®^ 64R centrifuge, Beckman Coulter, Palo Alto, CA, USA) for 10 min. The lipid pellet was extracted using an established volume of protein extraction solution (46% water, 42% isopropanol, 12% acetonitrile, 0.1% TFA), prior to HPLC injection (200 μL in the case of F98, 1.5 mL in the case of RBCs).

##### 2.3.6.3. Drug Content

Drug percentage recovery was calculated as the ratio between the real and theoretical drug content in the nanoemulsions. The drug content was assessed by HPLC, after extraction from nanoemulsions. In the case of doxorubicin and irinotecan, this was achieved via the 1:2 dilution of the samples with acetic acid and acetonitrile, respectively, followed by centrifugation at 14,300× *g* (MPW55, Medical Instruments, San Lazzaro di Savena, Italy), to discard the lipids. For cisplatin, however, 50 μL of nanoemulsion was diluted in 1 mL normal saline, to displace the HIP, and shacked with 1 mL chloroform to discard the lipid. The mixture was then centrifuged at 1540× *g* for 5 min (Rotofix 32 centrifuge, Hettich, Tuttingen, Germany), and 800 μL of the water phase underwent the derivatization reaction, prior to HPLC injection.

Percentage entrapment efficiency (EE%) was calculated as the ratio between the drug content in the oily droplets, isolated by centrifugation at 62,000× *g* (Allegra^®^ 64R centrifuge, Beckman Coulter, Palo Alto, CA, USA), and that in the whole nanoemulsion (oil + water). To this aim, in the case of doxorubicin and irinotecan, the drug was extracted from the oily pellet using a 1:2 water/acetic acid or water/acetonitrile mixture, respectively, whereas, for cisplatin, the drug content in the oily phase was estimated as the difference between the total and that observed in the water phase, which was determined by HPLC after derivatization.

##### 2.3.6.4. Drug Release

The release of the three drugs from the nanoemulsions was estimated using the dialysis bag (14,000 Da cut-off, Sigma-Aldrich, St. Louis, MO, USA) method. Free drugs were used as controls. Suitable amounts of the nanoemulsions were diluted in water up to 3 mL and inserted into a dialysis bag. The beakers in which the dialysis bags were immersed contained 30 mL of water-based release media; distilled water for doxorubicin, citrate buffer 0.01 M pH = 4.5 for irinotecan in order to avoid the lactone/hydroxyacid transition, and normal saline for cisplatin in order to displace AOT and reconstitute the parent drug from its prodrug. At scheduled times, 500 μL of the release media were collected and drug content was estimated using different methods; spectrofluorimetry for doxorubicin and irinotecan, HPLC after derivatization for cisplatin. Drug release was expressed as percentage of the total.

#### 2.3.7. Immunofluorescence

Immunofluorescence studies were performed via staining with specific antibodies; E-cadherin on F98 cell-based and CD47 on RBC-based formulations. To this aim, in separate experiments, 200,000 F98 cells (suspended in 0.2 mL of phosphate-buffered saline—PBS), 100 μL of F98 CMF suspension and 100 μL of F98 CMF-wrapped IL were incubated for 1 h at room temperature with 0.5 μg/mL CD324 (E-Cadherin) monoclonal antibody (DECMA)—Alexa Fluor™ 488, then centrifuged for 10 min (96× *g* for cells, 18,000× *g* for CMF and 62,000× *g* for IL), and re-suspended in 0.2 mL PBS. In a similar way, 1,000,000 purified rat RBCs (suspended in 1 mL of PBS), 100 μL of RBC CMF suspension and 100 μL of RBC CMF-wrapped IL underwent the same procedure as F98, using 0.5 μg/mL CD47/MER6 polyclonal antibody, FITC conjugated. Afterwards, a small drop of sample from the suspension was placed on a glass for fluorescence and observed using microscopy (DM2500, Leica Microsystems, Wetzlar, Germany, equipped with a Moticam 480 camera, Motic, Barcelona, Spain).

#### 2.3.8. Analytical Methods

##### 2.3.8.1. High Performance Liquid Chromatography (HPLC)

The HPLC analysis of doxorubicin was carried out using a literature method [21]. The HPLC analysis of irinotecan was carried out using a Chromsystems C18 12.5 × 5 mm column. The HPLC system was composed of a YL9110 quaternary pump, a YL9101 vacuum degasser and a YL9160 photo diode array (PDA) detector, linked to YL-Clarity software for data analysis (Young Lin, Hogyedong, Anyang, Korea). The gradient was performed between eluent A (phosphate buffer 0.05 M pH = 6.0 containing 1.1 mg/mL 1-heptanesulfonic acid) and eluent B (acetonitrile) [24]; 0 min: 90% A; 4 min: 90% A; 9 min: 60% A; 13 min: 60% A; 14 min: 90% A; 15 min: 90% A. The flow rate was set to 1 mL/min. The UV-visible detector was set at λ = 370 nm, and at λ = 220 nm for cell-internalization studies. The retention time of irinotecan was 11.5 min. SN-38 and camptothecin were used as internal standards. The lactone-to-hydroxyacid transitions of irinotecan, SN-38, and camptothecin were carried out by diluting the samples in acetonitrile and 0.1 M NaOH, and maintaining them at room temperature for 1 h. The retention times of the internal standards were as follows: camphtotecin hydroxyacid 7.9 min, SN-38 hydroxyacid 8.0 min, irinotecan hydroxyacid 10.0 min, camphtotecin lactone 10.5 min, SN-38 lactone 10.8 min, irinotecan lactone 11.9 min.

The HPLC analysis of cisplatin was carried out after derivatization [25], according to a literature method [26]. The reverse phase (RP) HPLC analysis of CMF proteins was carried out according a literature method [17].

##### 2.3.8.2. Spectrofluorimetry

In release studies, doxorubicin and irinotecan were quantified using a Shimadzu RF-551 spectrofluorometer (Shimadzu, Tokyo, Japan): λ_exc_ = 480 nm, λ_em_ = 555 nm for doxorubicin; λ_exc_ = 370 nm, λ_em_ = 430 nm for irinotecan.

#### 2.3.9. hCMEC/D3 Studies

hCMEC/D3, cultured as reported above, were seeded at 100,000/well in a collagen-coated polytetrafluoroethylene (PTFE) membrane Transwell (Corning Life Sciences, Chorges, France) device (6.5 mm insert diameter, 3 μm pore size) and grown for 4 days up to confluence. Permeability of 0.15 mg/mL FITC-dextran, taken as a parameter of paracellular transport across hCMEC/D3 monolayer, was measured spectrofluorimetrically (λ_exc_ = 490 nm, λ_em_ = 540 nm) with a multilabel plate reader (Victor3 1420, Perkin Elmer, Waltham, MA, USA), in order to assess the monolayer’s integrity.

At day 4, the receiving medium in the basal chamber was replaced with 3-day F98 cell culture supernatant, which should contain specific F98 chemo-attractant factors. Then, selected doxorubicin loaded formulations were dropped in the apical chamber at a concentration of 2.8 μg/mL. After 1, 3, and 6 h, the medium in basal chamber was collected and doxorubicin concentration was measured spectrofluorimetrically (λ_exc_ = 490 nm, λ_em_ = 615 nm), using a multilabel plate reader (Victor3 1420, Perkin Elmer, Waltham, MA, USA), owing to a previously set calibration curve (*n* = 5).

#### 2.3.10. F98 Cell Studies

##### 2.3.10.1. Invasion Assays with F98 CMF-Based Formulations

The invasion assay was used to demonstrate the ability of F98 CMF-wrapped IL to target the tumour. For this purpose, the formulations were used as a chemo-attractant stimulus, i.e., to induce the migration of F98 rat glioma cells from the upper to the lower compartments of a Boyden chamber (BD Biosciences, San Jose, CA, USA), separated by a 5 μm porous membrane treated with 50 µg/mL Matrigel^®^ (BD Biosciences, San Jose, CA, USA). A total of 2000 F98 cells per well were seeded in the upper compartment (in quadruplicate), while the various chemo-attractant factors, appropriately diluted in medium, were placed in the lower compartment for comparison purposes:

- FBS 20%, positive control
- F98 CMF diluted 1:5 (dilution range: 1/500–1/10,000)
- F98 CMF-wrapped IL (dilution range: 1/500–1/10,000)
- IL, internal control (dilution: 1/500)

After 6 h, the membrane was washed with PBS, the cells were fixed with methanol and subsequently stained with crystal violet. Invading cells were then counted under an inverted microscope (DM750, Leica Microsystems, Wetzlar, Germany). Data were expressed as percentage invasion of F98 cells in the absence of chemo-attractant factors (100% control) (*n* = 5).

##### 2.3.10.2. Flow Cytometry Cellular Uptake of 6-CUM Labelled Nanoemulsions

The cytofluorimetric internalization of 6-CUM-labelled nanoemulsions was investigated by incubating the formulations under study with F98 cells for 1 h at 37 °C with 5% CO_2_ in FBS-enriched culture medium, according to a literature methodology [17]. The incubation was carried out on cells suspended in tubes. 200,000 cells were used per condition, and the correct ratio between cells and labelled formulations was preliminarily established, to avoid saturation of cell uptake. Then, cells were centrifuged at 96× *g* (NF 200, Nuve, Ankara, Turkey) for 10 min, re-suspended in 0.5 mL phosphate-buffered saline (PBS) and analyzed with an Accuri C6 (BD Biosciences, Milan, Italy) flow cytometer (considering 10,000 events and medium flow rate). Any cell debris with low forward light scatter (FSC) and side light scatter (SSC) were excluded from the analyses. Untreated F98 cells were used as controls (Ctrl) and 6-CUM fluorescence was expressed as the integrated mean fluorescence intensity (iMFI), that is fluorescence-positive cells frequency per median fluorescence intensity.

##### 2.3.10.3. 2D Cell Studies with Drug-Loaded Nanoemulsions

The cytotoxicity of the drug-loaded formulations under study was evaluated using the MTT assay after 72 h of treatment [27,28] (*n* = 5). In the case of doxorubicin-based formulations, 24 h and 48 h treatments were also performed (*n* = 5) to simulate the conditions of organ-on-chip experiments.

In order to better elucidate the lactone/hydroxyacid transition of irinotecan and its metabolism by carboxylesterase, 24 h treatments were performed, in separate experiments, in 24-well plates at a concentration of 50,000 cells, with an irinotecan concentration of 24 μg/mL per well. After treatment, the medium was collected in an Eppendorf tube, frozen at −20 °C and subsequently analysed by HPLC. Cells were detached with 50 μL of trypsin and centrifuged at 2400 rpm (Fresco 21 Centrifuge, Heraeus Group, Hanau, Germany) for 5 min. The supernatant was removed and the pellet was frozen at −20 °C for subsequent HPLC analysis. Before HPLC analysis, the medium was diluted 1:1 with acetonitrile, centrifuged at 14,300× *g* (MPW55, Medical Instruments, San Lazzaro di Savena, Italy) for 5 min, while the cell pellet was extracted with 50 μL of acetonitrile and centrifuged at 14,300× *g* for 5 min.

Annexin V/propidium iodide (PI) assay, followed by flow cytometry analysis, was assessed to evaluate apoptosis on selected formulations (doxorubicin loaded). The F98 cell line was seeded in 6-well plates (300,000 cells/well). After 24 h, adherent and non-adherent cells were collected, washed in 1 × PBS, and stained with annexin V, conjugated to the FITC dye, and propidium iodide (PI), according to the manufacturer protocol. Cells were analyzed using a FACScan cytometer Accuri C6 (Becton Dickinson Italia, Milan, Italy) (*n* = 3).

##### 2.3.10.4. 3D Cell Studies Using Organ-On-Chip MIVO^®^ Technology with Drug-Loaded Nanoemulsions

MIVO^®^ technology (React4life, Genoa, Italy) was used to reproduce the nanoemulsion-based drug-targeting phenomenon in a three-dimensional (3D) *in vitro* flow system. A peristaltic pump circulated the culture medium through a series of tubes across the basal chamber of a Transwell^®^, while, in the apical chamber, F98 cells were cultured in 3D mode using alginate beads (React4life). Briefly, 100 µL alginate was mixed with 500,000 F98 cells and then resuspended in 100 µL of CaCl_2_-enriched DMEM; 18 µL of this cell suspension was then gently transferred, using a micropipette, into a plate containing the cross-linking agent (CaCl_2_ based). Alginate spheroids containing 30,000 cells were formed after 1 min of gentle shaking. The spheroids were then washed and transferred to a 48-well plate in an incubator with 5% CO_2_ for 24 h, before being placed in the apical chamber of a Transwell^®^. In separate experiments, the culture medium was placed in the circuit, in the absence or in the presence of the formulations under study, at 2 mL/min flow (approximately 9 rpm with 2 mm diameter tubes).

After 24 h of treatment, cell internalization and cytotoxicity were evaluated in separate experiments. In the former case, drug internalization within cells was evaluated as follows; the spheroids were placed in Petri dishes and contacted with DAPI (1 μg/mL in normal saline) for approximately 30 min, then washed with PBS, placed on a glass slide, gently compressed with a cover slide and observed under fluorescence microscopy (DM2500, Leica Microsystems, Wetzlar, Germany, equipped with a Moticam 480 camera, Motic, Barcelona, Spain). In a separate experiment, a clonogenic assay on de-alginated cells was performed to assess cytotoxic activity. Briefly, the spheroids were transferred to an Eppendorf tube with 200 µL of de-cross-linking solution (tri-sodium citrate-based), kept in an incubator for 30 min and then centrifuged twice at 1400× *g* (Fresco 21 Centrifuge, Heraeus Group, Hanau, Germany) for 7 min. The obtained cell pellet was transferred to a 6-well plate and then left growing with fresh medium for 5 days, before adherent cells were counted under a DMIL microscope, coupled with a DFC450 C camera (Leica Microsystems, Wetzlar, Germany) (*n* = 3).

#### 2.3.11. Statistical Analysis

One-way ANOVA analysis was performed using GraphPad InStat software (San Diego, CA, USA), and this was followed by the Bonferroni multiple comparison post-test to evaluate differences in the experimental groups. Values of *p* ≤ 0.05 were considered statistically significant.

## 3. Results

### 3.1. Physico-Chemical Characterization of Nanoemulsions

The CMF-wrapping process was followed step-by-step by optical microscopy (Figure S1). Rat RBCs were used as a model, in addition to F98 glioma cells, since they are present in high concentration in the blood, and thus also allow microscopy visualization to be optimized by means of FITC labelling. In optical microscopy the morphology of the nanostructures is similar whether coated with F98 or RBCs, although they can be detected more easily with the RBC coating. Indeed, CMFs are round-shaped vesicles that form donut-shape structures upon gentle mixing with IL. Extrusion with 220 nm filters under a sterile hood is a fast and easy way to wrap oily droplets in CMFs, with the process resulting in homogeneous sizes and sterile formulations (Table 1). Of note, reported DLS data for CMF showed a high polydispersity, especially in the case of F98. This might be ascribed to the presence of residual large-sized impurities in the CMF suspension, which heavily influence the DLS unimodal evaluation, which is based on the scattering intensity only. Indeed, based upon raw DLS data (Figure S2), multimodal size distribution suggests that in the case of F98 CMF a lower mean particle size (188 nm) might be hypothesized as the major component in number of particles in the CMF suspension.

Purification from excess proteins that overcome the loading limit of the oily droplet surface is achieved via ultracentrifugation and re-suspension. However, RBCs and F98 show different behaviour here, because of the higher protein content of RBCs, which is proportional to cell count. Indeed, the majority of F98 CMF proteins, but only a small amount of those from RBC CMFs, are layered on the oily droplets (Table 1). This is made visually clear in the FITC-labelled RBCs, whereas fluorescence is hard to detect in CMF-wrapped IL, and, even then, only in the case of aggregated droplets (Figure S1). The Zeta potential of CMFs is less negative than that of IL, with this likely being due to the protein content of these vesicles. Therefore, the Zeta potential of (unloaded) CMF-wrapped IL is at an intermediate value between those of (unloaded) IL and CMFs (Table 1).

**Figure 1:**
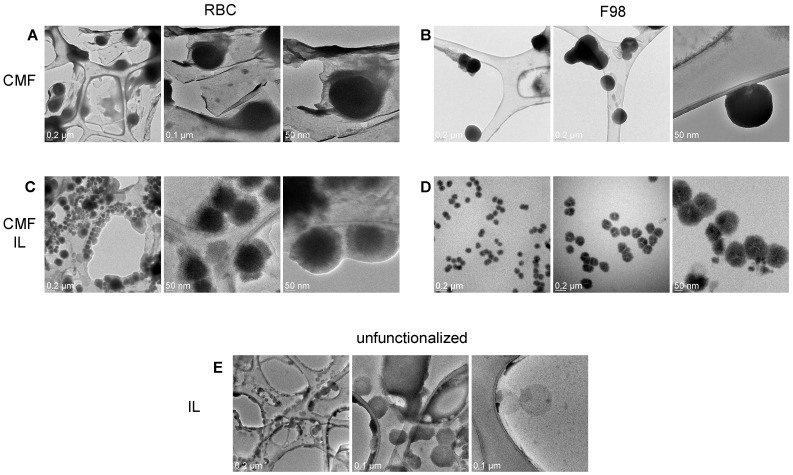
TEM of RBC and F98 CMF, and CMF-wrapped IL. (**A**): RBC CMF; (**B**): F98 CMF; (**C**): RBC CMF-wrapped IL; (**D**): F98 CMF-wrapped IL; (**E**): unfunctionalized IL (from [28]). Three images with different magnifications reported for each panel. Abbreviations: CMF: cell membrane fragments; IL: Intralipid^®^ 10%; RBC: red blood cells; TEM: transmission electron microscopy.

TEM allowed us to appreciate the morphological differences in the formulations under study (Figure 1).

Indeed, while F98 CMFs appear as highly contrasted, circular and regular structures, a halo, which can likely be attributed to surface proteins, is present around RBC CMFs. Differences can also be observed in uncoated and CMF-wrapped IL. A low-contrast shell, likely attributable to rich protein content, surrounds the oily droplet in RBC CMF-wrapped IL, while a compact coating is present in F98 CMF-wrapped IL.

In this work, F98 CMF-wrapped IL were used as a versatile platform to load three chemotherapeutic drugs (doxorubicin, irinotecan, cisplatin), with a view to their use in homotypic targeted delivery to glioma. Doxorubicin was also loaded in RBC CMF-wrapped IL for comparison purposes. In addition, uncoated IL was loaded with the three drugs to act as a control for the subsequent cell studies (Table 1). Since the three drugs are water soluble, they were loaded within the IL lipid matrix in the form of HIPs with AOT. This process did not involve any chemical modification of the parent drugs and no new chemical entity was synthesized; rather, water-insoluble salts (doxorubicin-AOT, irinotecan-AOT) and prodrugs (cisplatin–AOT) were formed (Table S1), which can be displaced by endogenous components in the physiological medium [29].

Drug loading did not affect droplet size in a relevant manner. However, it is hard to establish a correlation between drug loading and Zeta potential due to the multiple charge interactions that occur at the surface of the HIP-loaded formulations. Drug recovery was affected by the efficiency of the micropipette mixing used to load the drug into the nanoemulsions, and was highest for doxorubicin–AOT. Drug EE% in uncoated IL was different for the HIPs studied (cisplatin–AOT > doxorubicin–AOT > irinotecan–AOT). It is worth noting that F98 CMF wrapping increased drug EE% for all three drugs, and this increase was particularly substantial for irinotecan–AOT and cisplatin–AOT (Table 1). Optical microscopy shows that no insoluble crystals were present in the nanoemulsions (Figure S3). The fluorescence mode highlighted the fact that higher drug EE%, such as in the case of doxorubicin, correlates with dotted fluorescence around the oily droplets. Moreover, in the case of F98 CMF-wrapped IL, increased drug fluorescence is located around the oily droplet, matching the CMF coating. Given the size limit of optical microscopy, this phenomenon is more evident in the case of over-sized droplets. The drug EE% values, in turn, are reflected in the release profiles of the formulations under study (Figure S4). Free drugs, used as controls, gave faster release, indicating that the dialysis membrane was not the limiting step; on the contrary, free ion pairs displayed the slowest release, which was most likely due to slow dissolution in the release medium. Drug release from nanoemulsions depended upon HIP oil/water partition, and slower release correlated well with higher drug EE%. In fact, relevant differences in F98 CMF-wrapped and uncoated IL were noted in the case of irinotecan–AOT and cisplatin–AOT.

### 3.2. Immunofluorescence

**Figure 2:**
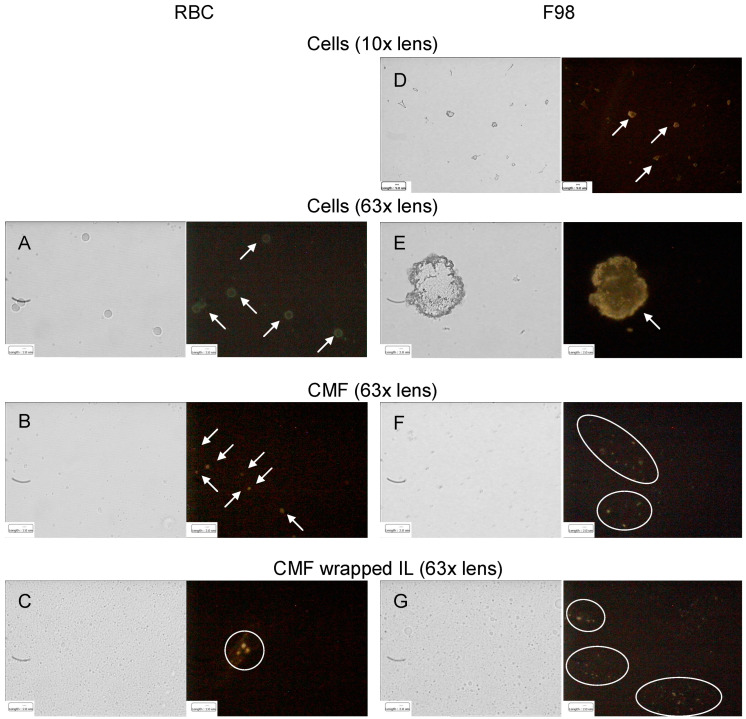
Immunofluorescence of RBC (CD47) and F98 cell (E-cadherin) membrane protein markers throughout the IL CMF wrapping process. (**A**): RBC; (**B**): RBC CMF; (**C**): RBC CMF-wrapped IL; (**D**): F98 cells (lower enlargement); (**E**): F98 cells (bigger enlargement); (**F**): F98 CMF; (**G**): F98 CMF-wrapped IL. Normal light and fluorescence images reported for each panel. In fluorescence images corpuscolate and dotted fluorescence are indicated by arrows and circles, respectively. Abbreviations: CMF: cell membrane fragments; IL: Intralipid^®^ 10%; RBC: red blood cells.

The hypothesized homotypic targeting performed by F98 CMF-wrapped IL relies upon the retention of specific tumour-adhesion proteins on the coated IL. Given that E-cadherin is the most expressed of these proteins in glioma [30,31,32], immunofluorescence was used to assess its presence throughout the wrapping process. In a parallel experiment, CD47, an RBC membrane protein responsible for immune escape [14,15], was also tracked during the RBC CMF wrapping process (Figure 2).

It is worth noting that, according to the experimental data, both E-cadherin and CD47 were highly expressed on the respective parent cells. Moreover, specific fluorescence was observed, as dots around the oily droplets, in vesicular CMF and in CMF-wrapped IL, confirming protein retention in the coated nanoemulsions.

### 3.3. Assessment of CMF Chemo-Attractant Properties

**Figure 3:**
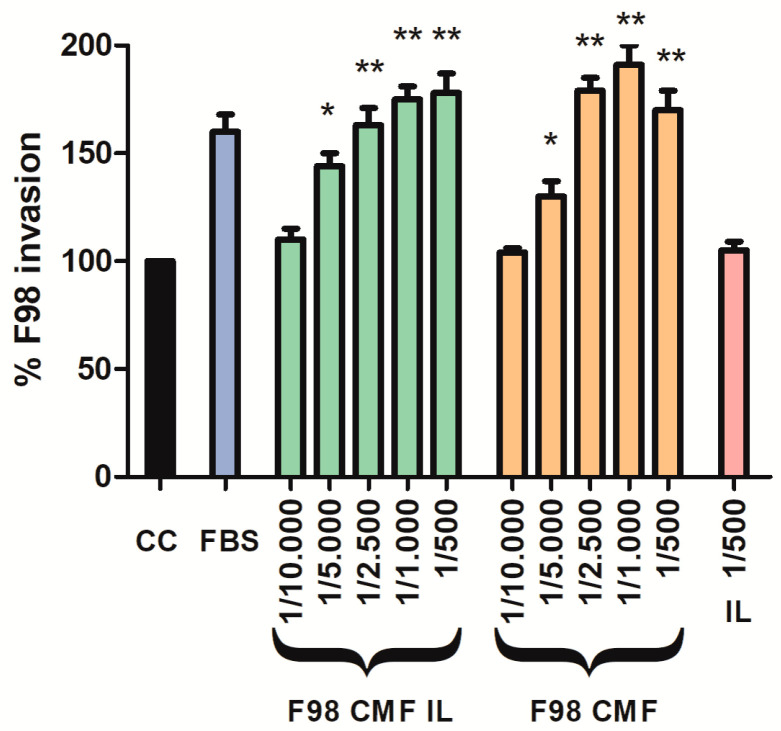
Induction of parent cell invasion by F98 CMF. Employed dilutions are reported on the *x* axis. Abbreviations: CC: control (without any chemo-attractant); CMF: cell membrane fragments; FBS: fetal bovine serum 20%. Statistical analysis: * *p* < 0.05 vs. CC; ** *p* < 0.01 vs. CC.

Given the assessed retention of E-cadherin, which is an adhesion protein, on the surface of F98 CMF-wrapped IL, suitable 2D cell models were exploited to investigate the chemo-attractant properties of the formulations towards F98 cancer cells, upon which the homotypic targeting mechanism relies. To this aim, an invasion assay was performed using the Boyden chamber; FBS, which is the default chemo-attractant often used in similar experiments, was compared to F98 CMF and F98 CMF-wrapped IL. Significantly, the formulations under study exerted relevant chemo-tactic efficacy, comparable to that of FBS, in a dose-dependent manner (Figure 3).

**Figure 4:**
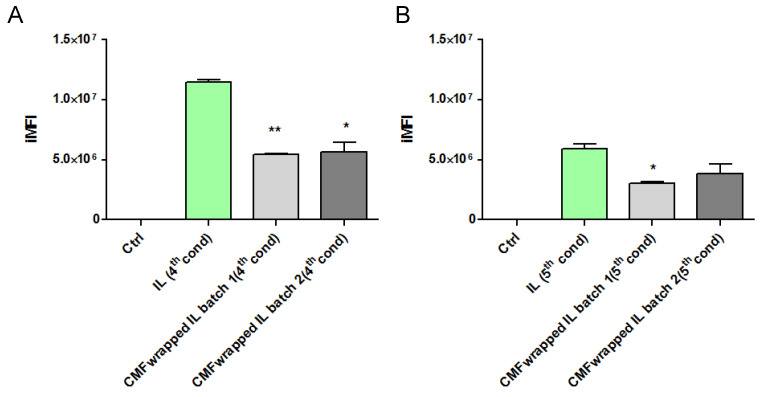
F98-cell internalization of 6-cum-labelled unfunctionalized and F98 CMF-wrapped IL using flow cytometry. (**A**) Cell/lipid ratio as condition 4; (**B**) cell/lipid ratio as condition 5. Abbreviations: 6-cum: 6-coumarin; CMF: cell membrane fragments; IL: Intralipid^®^ 10%; iMFI: integrated mean fluorescence intensity. Statistical analysis: * *p* < 0.05 vs. IL; ** *p* < 0.01 vs. IL.

Therefore, in a subsequent experiment, uncoated and F98 CMF-wrapped IL were loaded with the fluorescent probe 6-CUM, which is capable of efficiently labelling the lipid matrix (Figure S5), in order to perform F98 cell-internalization experiments via flow cytometry [17]. Preliminarily, 6-CUM-labelled IL was employed to optimize the F98 cell/lipid ratio and thus avoid the saturation of cell entry mechanisms (Figure S6). Conditions 4 and 5 were then selected to investigate the effect of F98 CMF wrapping on cell internalization (Figure 4). Experiments were performed with two different batches of F98 CMF-wrapped IL.

Unexpectedly, F98 CMF wrapping led to a decrease in IL internalization within cells, in all tested conditions and batches. A potential explanation for this phenomenon can be hypothesized. While flow cytometry evaluates the internalization of fluorescently-labelled colloids in the cytoplasm, the higher expression of E-cadherin in F98 CMF, compared to other types of cancer cells [17], can promote the adhesion processes of IL to the cell membrane. With this in mind, E-cadherin maintains cell–cell junctions by continuously forming short-lived adhesive dimers, and endocytosis is the main route for their dissociation [33]. Indeed, in the absence of endocytosis, these dimers are very stable from a chemical standpoint. It has also been demonstrated that, when E-cadherin is chemically grafted onto beads, dimers are formed between the proteins that belong to the cells and beads [34]. Therefore, it might be hypothesized that, in the presence of F98 CMF-wrapped IL, stable cell–CMF adhesive dimers are formed on the cell surface, probably preventing the endocytosis of both the E-cadherin associated with cells and that associated with the CMF-wrapped IL droplets.

### 3.4. Cell Studies with Drug-Loaded Nanoemulsions

**Figure 5:**
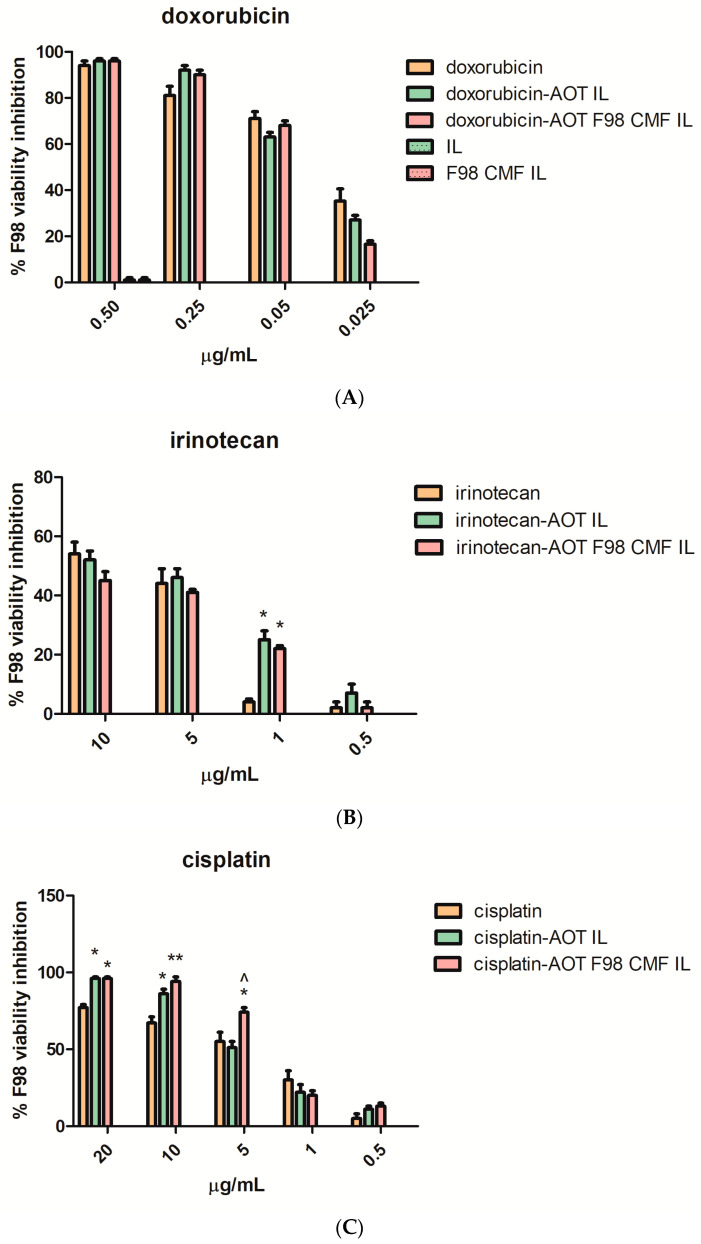
MTT assay at 72 h, performed on F98 cells, of the free drugs, unfunctionalized drug-loaded IL, and drug-loaded F98 CMF-wrapped IL. (**A**) Doxorubicin; (**B**) irinotecan; (**C**) cisplatin. Abbreviations: AOT: sodium docusate; CMF: cell membrane fragments; HIP: hidrophobic ion pair; IL: Intralipid^®^ 10%; MTT: 3-(4,5-dimethylthiazol-2-yl)-2,5-diphenyltetrazolium bromide. Statistical analysis: * *p* < 0.05 vs. free drug; ** *p* < 0.01 vs. free drug; ^ *p* < 0.05 vs. HIP-loaded IL.

As CMF-wrapped IL works as a versatile platform for the loading of several drugs, in the form of HIPs (Table 1), the cytotoxicity of the drug-loaded formulations against F98 cells, cultured in 2D mode (MTT assay) after 72 h treatment (Figure 5) was studied. In these studies, the free drugs, dissolved in DMSO, were compared with the corresponding HIPs, loaded either in uncoated IL or in F98 CMF-wrapped IL, to establish a dose-response curve. The solution of free cisplatin in DMSO was stored at −20 °C, for cytotoxicity studies, to avoid the drug inactivation that may occur via the formation of transplatin–DMSO adducts [35,36,37,38].

In Figure 5A, regarding doxorubicin loaded formulations, it is shown that the unloaded nanoemulsions exerted negligible cytotoxicity, regardless of F98 CMF coating. Therefore, unloaded formulations were no longer included in the studies involving irinotecan and cisplatin-loaded formulations (Figure 5B,C). The cytotoxic activity of the free drugs was maintained in the formulations under study. A significant increase in cytotoxicity was noted, in the case of cisplatin, after loading in IL and after CMF wrapping.

The effect of IL loading and F98 CMF wrapping on irinotecan’s lactone/hydroxyacid transition and SN-38 conversion by carboxylesterases was investigated, in a separate series of experiments, since the phenomena may affect cytotoxicity. Indeed, the lactone/hydroxyacid transition is responsible for drug inactivation, while SN-38 conversion by carboxylesterases is responsible for the formation of the active metabolite of the irinotecan prodrug [24]. Analyses were performed after 24 h of treatment, in order to prevent massive cell death, which would hamper the correct evaluation of internalization, using a slightly higher drug dose than in the MTT experiments, in order to facilitate HPLC detection. While the irinotecan lactone/hydroxyacid transition was unaffected by the vehicle (Figure S7), plain SN-38 lactone was isolated in only a few replicates at low amounts, because its low water solubility hampers detection above the nanomolar range [39,40,41]. This probably means that a correct evaluation of SN-38 conversion should be performed at very low drug concentrations, which would not be predictive of MTT conditions. Nonetheless, a higher SN-38 hydroxyacid/irinotecan ratio was detected after the IL and F98 CMF-wrapped IL treatments (Figure S7). Unlike SN-38 lactone, SN-38 hydroxyacid is inactive, and, moreover, can originate from both SN-38 lactone and inactive irinotecan hydroxyacid. Yet, given that the irinotecan lactone/hydroxyacid transition was unchanged after both the drug loading in IL and CMF-wrapped IL experiments, a higher SN-38 hydroxyacid/irinotecan ratio might indirectly indicate increased SN-38 lactone conversion. Interestingly, this matches the increased irinotecan internalization in F98 cells, where carboxylesterase metabolism should take place (Figure S7). However, this phenomenon only resulted in a significant cytotoxicity increase, compared to the free drug, at a low dose (1 μg/mL) (Figure 5).

To assess the contribution of apoptosis in the observed MTT value reductions on selected formulations (doxorubicin loaded), the annexin V/PI assay was performed. F98 cells were treated with F98 CMF-wrapped IL (F98 CMF-IL), free doxorubicin, doxorubicin–AOT-loaded IL, and F98 CMF-wrapped doxorubicin–AOT-loaded IL, respectively, at a concentration of 0.5 μg/mL doxorubicin. After cytofluorimetric analysis of the annexin V/PI staining, necrotic (annexin V−/PI+), early (annexin V+/PI−), and late apoptotic (annexin V+/PI+) cells were individuated. F98 cells were affected by the treatments. Indeed, higher levels of annexin V+, corresponding to both early and late apoptosis, were observed in F98 cells treated with doxorubicin-loaded formulations with respect to those treated with the blank vehicle (F98 CMF-wrapped IL). Moreover, a significantly higher apoptotic cell percentage was observed after treatments with doxorubicin–AOT-loaded IL, both unfunctionalized and F98 CMF-wrapped, with respect to free doxorubicin (Figure S8). Finally, a significant increase in PI positive necrotic cells was observed for doxorubicin (10.29 ± 0.94%) and doxorubicin–AOT-loaded IL (8.90 ± 0.55%) treated cells—but not for doxorubicin–AOT F98 CMF-wrapped IL (5.90 ± 1.97%)—with respect to F98 CMF- wrapped IL-treated cells, taken as controls (3.50 ± 0.63%).

The BBB’s overcoming potential of the proposed approach was tested on hCMEC/D3 model, which constitutes an FITC–dextran almost impermeable cell barrier (Figure S9A). Doxorubicin was assumed as the model drug, owing to its intrinsic fluorescence. Loading in nanoemulsions allowed a faster doxorubicin depletion from the Transwell apical chamber after a 3 h exposure, likely due to uptake by hCMEC/D3 monolayer (Figure S9B). However, internalization in the endothelial cells is not sufficient to guarantee BBB overcoming. Of note, in the hCMEC/D3 model, CMF wrapping seems to slightly increase the translocation of doxorubicin from the monolayer to the basolateral chamber; this phoenomenon can be appreciated after a 6 h exposure (Figure S9C). Nonetheless, this 2D model, which should resemble the intact BBB, is only partially capable to recapitulate the brain tumour microenvironment, which, besides, is characterized by a leaky BBB, the so-called blood–brain-to-tumour barrier (BBTB), which is more permeable to nanocarriers.

**Figure 6:**
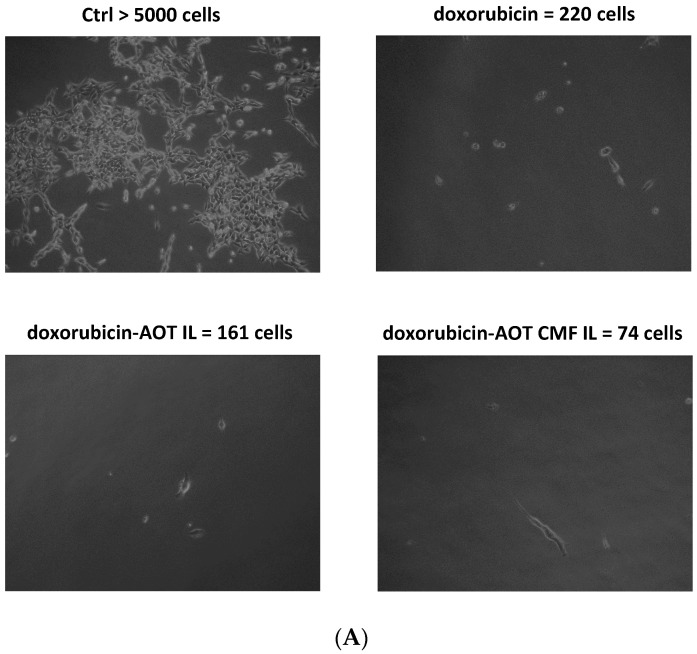

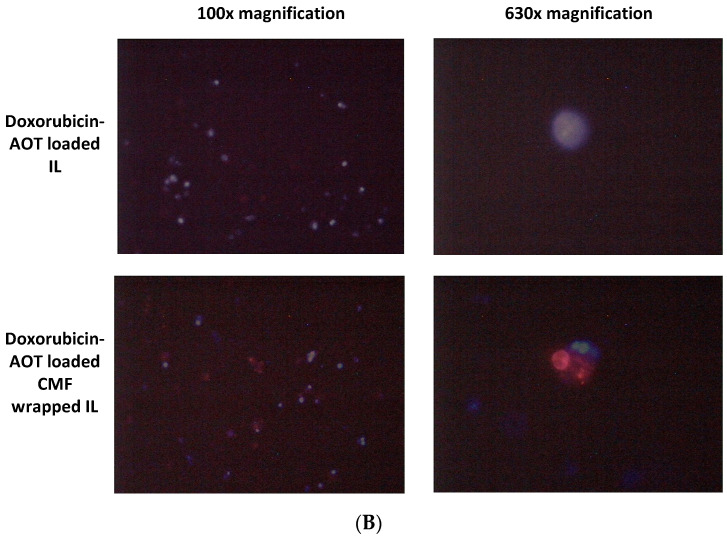
MIVO^®^ studies with 3D alginate included F98 cells, treated with doxorubicin–AOT-loaded, unfunctionalized, and F98 CMF-wrapped IL. (**A**) Clonogenic assay of de-alginated F98 cells from Transwell^®^ upper chamber; numbers refer to total well adherent cell counting. Reported images are representative of a sample area. (**B**) Fluorescence microscopy of F98 cells cultured in the alginate beads from Transwell^®^ upper chamber (red channel: doxorubicin; blue channel: DAPI). Abbreviations: AOT: sodium docusate; CMF: cell membrane fragments; ctrl: untreated control; DAPI: 4′,6-Diamidino-2-phenylindole; IL: Intralipid^®^ 10%.

Therefore, the promising results achieved in 2D cell models drove us to investigate the potential of F98 CMF-wrapped IL as a drug delivery system in 3D cell models, using alginate bead-based matrixes with organ-on-chip MIVO^®^ technology. Doxorubicin–AOT-loaded formulations were selected for this objective as they allow both cytotoxicity and cell internalization to be evaluated in the same set of experiments, thanks to clonogenic assays and fluorescence optical microscopy. Indeed, although doxorubicin and irinotecan are both fluorescent drugs, preliminary studies led to the former being selected due to its better fluorescence signal-to-noise ratio. Therefore, the cytotoxicity of doxorubicin-based formulations was preliminarily assessed using MTT assays after 24 and 48 h of treatment, in order to optimize conditions and doses for MIVO^®^ experiments (Figure S10). It is important to note that, at treatment timeframes lower than 72 h, doxorubicin–AOT-loaded IL (but not the F98 CMF-wrapped formulation) shows significantly higher cytotoxicity at a 0.25 μg/mL dose. However, the lowest dose tested (0.05 μg/mL) was selected for the MIVO^®^ experiment, to better appreciate the effect of drug loading in IL and CMF wrapping on cytotoxicity (Figure 6).

After 24 h of the MIVO^®^ experiment, de-alginated F98 cells were cultured for 5 days in drug-free medium. After this period, untreated conditions were almost confluent, while doxorubicin-based treatments were able to inhibit cell growth to a different extent: doxorubicin–AOT CMF IL > doxorubicin–AOT IL > doxorubicin. In 2D mode (MTT assay), no significant differences were noted in the formulations at 0.05 μg/mL after 24 h of treatment. It might therefore be hypothesized that the MIVO^®^ results were affected by the capability of the drug-delivery systems to overcome the barriers of the 3D model. In particular, the considerable cell-growth inhibition found with CMF-wrapped IL may be related to the occurrence of CMF–cell chemo-tactic interactions, as previously established in the invasion assay. This is confirmed by the increased fluorescence of the doxorubicin internalized in F98 cells when CMF-wrapped IL was used for the treatments (Figure 6).

## 4. Discussion

CMFs have recently gained scientific attention as they are endowed with several interesting properties that derive from the cell that they originate from [13]. These features include immune escape for RBC-derived CMFs [9,14,15], and targeting capability in the case of cancer cell origins. These features stem from several mechanisms but are mainly due to the expression of adhesion proteins on cancer cell surfaces [12,16,17].

High-grade gliomas have poor prognosis due to fatal relapses after surgical resection, in the absence of an efficient chemotherapy. The main limit of chemotherapy is the BBB, which significantly limits the access of potential drug candidates to the brain [1]. IL has been recently purposed for cytotoxic drug delivery against glioma [42]. In this experimental work, the BBB crossing potential of IL-loaded drugs was demonstrated by means of *in vitro* hCMEC/D3 models, as well as the enhancing effect of CMF wrapping, in agreement with literature data [43]. Of course, an *in vivo* setting is endowed with more critical concerns, including metabolism. Animal experiments exceed the aims of this work, yet, within this context, some encouraging literature evidence assesses the potential of IL as a drug delivery system to the brain. Indeed, although after IV administration a modest amount of IL is reported to permeate the BBB [44,45], IL has been frequently used to treat life-threatening toxicity, involving the central nervous system, due to local anesthetics, benzodiazepines, antidepressants, and other lipophilic poisoning agents [46,47,48]. Furthermore, likely due to the BBB permeability of its lipid constituents [49,50], IL has been recently purposed for the treatment of Alzheimer’s disease, which is associated with an altered BBB [51]. Of note, recent work aimed to compare its biological fate after administration by different routes, fluorescently-labeled IL showed a discrete brain accumulation by IV. [52]. Also noteworthy, the BBB is altered in glioma due to uncontrolled tumour growth and increased vascularization, leading to the so-called BBTB, which is more permeable than the BBB, and a potential subject for targeted nanocarrier-mediated drug delivery, which can occur via multiple potential mechanisms [53,54].

Since E-cadherin, a model for cell–cell adhesion proteins, is highly expressed in glioma [30,31,32], glioma-derived CMFs could work as a suitable agent for the homotypic targeting of nanocarriers. Several works in the literature propose wrapping innovative nanocarriers with CMFs. However, most preclinical nanocarriers suffer clinical failure due to efficacy and/or safety issues. However, injectable nanoemulsions have been in clinical usage for several decades, both for total parenteral nutrition and drug delivery [7,8]. Moreover, being deformable and filterable through a 220 nm syringe filter, CMF coating and sterilization can be merged into a single step to give a targeted nanoemulsion with a homogeneous size distribution [17].

Selected drug candidates have been loaded within a targeted nanocarrier to assess its potential as a versatile platform for drug delivery. Given that a considerable number of anti-cancer chemotherapeutics are water-soluble salts, entrapment within a lipid matrix of nanoemulsions was promoted via the formation of HIPs with AOT. This is a solvent-free technique that does not imply any chemical synthesis or new chemical entity being formed, but is solely based on electrostatic interactions between the drug and a surfactant with an opposite charge. Once the targeted nanoemulsion drives its cargo into the tumour, the parent drug is released from the HIP, owing to interactions with physiological components [29]. CMF-wrapped IL has proven its ability to effectively load all three drugs, while either maintaining or increasing their cytotoxic and apoptotic activity in 2D cultured cell models.

Moreover, this work presents a considerable amount of evidence to account for the cancer cell adhesion properties of F98 CMF-wrapped IL, in particular an invasion assay. However, clinical failure is often caused by an excessive reliance on simplistic pre-clinical *in vitro* models, while *in vivo* tumour models are expensive and associated with ethical issues [55]. For this reason, novel 3D tumour spheroids have recently gained more attention for their use in preliminarily tests, prior to animal experiments, and as alternative models. Indeed, although still far from the *in vivo* scenario, they can recapitulate many physiological features by providing an artificial extracellular matrix, where cell–cell and cell–matrix interactions, cell motility and proliferation occur in a more predictive way, compared to cell monolayers [56,57,58]. In particular, alginate beads are considered an innovative tool with which to simulate a 3D tumour [59,60,61,62], as they allow specific CMF–cell adhesive interactions, such those underlying the homotypic targeting phenomenon, to be mimicked. Moreover, the MIVO^®^ micro-fluid system, one of the currently available organ-on-chip technologies, allows 3D cancer models and dynamic flow conditions to be merged, making the system suitable for recapitulating the effect of physiological blood flow on the survival and invasion of tumour cells [63,64,65], as well as on drug targeting and distribution.

Therefore, the promising results obtained with MIVO^®^ experiments are potentially predictive of *in vivo* behaviour. Moreover, the scalability, versatility and efficacy of the proposed approach are relevant advantages from a translational standpoint. Indeed, the development of a personalized nanomedicine technique can ideally be foreseen, in which patient biopsies after surgery serve as a source of CMF, allowing autologous administration of CMF-wrapped IL into the same patient. Nonetheless, although organ-on-chip technologies are a versatile tool with which to optimize the conditions for efficient tumour-drug delivery, they fail to reproduce the effect of *in vivo* drug biodistribution and metabolism. Unfortunately, this can only be evaluated by animal testing, which should be carefully selected to truly recapitulate the human setting [58]. The choice of the F98 rat glioma cell model, as the CMF source, is therefore not random, but preparatory to further animal studies. In fact, F98/Fischer rats are a syngeneic glioma model, implanted by stereotaxic surgery, that results in rapid and reproducible glioma growth in an immuno-competent environment [18,19], thus resembling human conditions. To this aim, preliminary cytotoxicity studies were also performed using doxorubicin–AOT-loaded IL, wrapped with CMF derived from rat RBCs, as a primary cell source (Figure S11). The similar behaviour of the CMF derived from F98 cells (syngeneic with Fischer rat) and primary rat RBCs accounts for the feasibility of the autologous-like administration of F98 CMF-wrapped IL to F98/Fischer models, which, in turn, would be predictive of personalized nanomedicine in humans.

## 5. Conclusions

In this experimental work, CMFs derived from F98 rat glioma cells have been used to wrap IL in order to engineer a functionalized nanocarrier that is suitable for homotypic targeting, with this nanocarrier being assessed using available 2D and 3D cell models. This approach, which is based on nanoemulsions used in clinics for several decades, proved to be scalable, efficient in terms of chemo-tactic/targeting ability and versatile, as capable of loading different drugs in the form of HIPs, which can be displaced in the target site, releasing the parent molecule. Therefore, undoubted translational advantages towards personalized nanomedicine can be foreseen, which will be properly addressed in future *in vivo* studies on the syngeneic F98/Fischer rat glioma model.

## Figures and Tables

**Table 1:** DLS, Zeta potential, protein content, (ion-paired) drug percentage recovery and EE% of the formulations under study: unfunctionalized and RBC- and F98-CMF-wrapped nanoemulsions; F98 and RBC CMFs. Abbreviations: AOT: sodium docusate; CMF: cell membrane fragments; DLS: dynamic light scattering; EE%: percentage entrapment efficiency; IL: Intralipid^®^ 10%; RBC: red blood cells.

	Proteins		Ion Pair	Drug			Size		Z Potential
	µg/mL	µg/mg Lipid	mg/mL	Dose (mg/mL)	EE%	% Recovery	nm	Polydispersity	mV
IL	-	-	-	-	-	-	265.2 ± 2.5	0.101	−47.9 ± 4.6
			Doxorubicin–AOT (0.5)	0.27	55.3 ± 4.4	110.1 ± 23.4	259.3 ± 2.6	0.051	−43.5 ± 2.9
			Irinotecan–AOT (2.0)	1.2	29.0 ± 1.8	62.6 ± 6.9	248.3 ± 5.9	0.085	−35.7 ± 3.0
			Cisplatin–AOT (7.4)	2.0	36.5 ± 6.9	68.2 ± 9.2	258.4 ± 2.2	0.131	−46.4 ± 3.6
F98 CMF	4.68 ± 0.02	-	-	-	-	-	2349.5 ± 1887.0	0.748	−17.5 ± 3.6
F98 CMF IL	2.04 ± 0.68	0.0204 ± 0.0096	-	-	-	-	254.8 ± 5.5	0.056	−25.2 ± 1.6
			Doxorubicin–AOT (0.5)	0.27	76.0 ± 6.8	95.9 ± 26.9	266.4 ± 3.5	0.088	−39.1 ± 4.7
			Irinotecan–AOT (2.0)	1.2	71.4 ± 7.14	74.3 ± 17.9	281.1 ± 2.6	0.056	−34.8 ± 7.2
			Cisplatin–AOT (7.4)	2.0	78.6 ± 1.3	69.0 ± 12.2	244.5 ± 1.8	0.005	−51.1 ± 9.1
RBC CMF	28419.64 ± 2273.57	-	-	-	-	-	698.1 ± 45.3	0.230	−6.1 ± 4.3
RBC CMF IL	93.21 ± 8.39	0.93 ± 0.093	-	-	-	-	266.2 ± 1.5	0.100	−22.5 ± 0.1
			Doxorubicin–AOT (0.5)	0.27	80	73.7	277.1	0.097	−32.7 ± 4.5

## Data Availability

The data presented in this study are available on request from the corresponding author.

## Supplementary Materials

The following supporting information can be downloaded at https://www.mdpi.com/article/10.3390/cells13070641/s1: Figure S1: Optical microscopy of IL wrapping process with RBC and F98 CMF. Paired fluorescence and normal-light images are reported for FITC-labelled RBC. Upper panel: cells; upper intermediate panel: CMF; lower intermediate panel: CMF + IL mix; lower panel: CMF/IL mix after extrusion and resuspension. Right panel: RBC; middle panel: FITC labelled RBC; left panel: F98 cells. 630× magnification. Abbreviations: CMF: cell membrane fragments; FITC: fluorescein isothiocyanate; IL: Intralipid^®^ 10%; RBC: red blood cells. Table S1: Ion-pair characterization. Percentage purity was calculated as the percentage ratio between measured and theoretical drug mg_eq_ for each mg ion pair. Abbreviations: AOT: sodium docusate; mg_eq_: mg equivalent. Figure S2: Raw DLS data of F98 (**A**) and RBC (**B**) CMF: autocorrelation curve; lognomal size distribution; MSD summary by number of particles. For MSD lecithin was hypothesized as the main component of CMF, with a refractive index = 1.459 (Bansal, N.; Truong, T.; Bhandari, B. Feasibility study of lecithin nanovesicles as spacers to improve the solubility of milk protein concentrate powder during storage. *Dairy Sci. Technol.* 2017, 96, 861–872, doi:10.1007/s13594-016-0307-0). Abbreviations: CMF: cell membrane fragments; DLS: dynamic light scattering; MSD: multimodal size distribution; RBC: red blood cells. Figure S3: Optical microscopy (florescence and normal light) of drug-(doxorubicin–AOT, irinotecan-AOT, cisplatin–AOT)loaded, unfunctionalized and RBC and F98 CMF-wrapped nanoemulsions. Upper panel: doxorubicin–AOT; middle panel: irinotecan-AOT; lower panel: cisplatin–AOT. Magnification 630X. Abbreviations: AOT: sodium docusate; CMF: cell membrane fragments; RBC: red blood cells. Figure S4: Drug (doxorubicin, irinotecan, cisplatin) release from doxorubicin–AOT, irinotecan–AOT, cisplatin–AOT-loaded nanoemulsions; right panel: doxorubicin; middle panel: irinotecan; left panel: cisplatin. Abbreviations: AOT: sodium docusate; CMF: cell membrane fragments. Figure S5: Fluorescence microscopy of 6-cum labelled nanoemulsions. Right panel: IL; left panel: F98 CMF-wrapped IL. Magnification 630X. Abbreviations: 6-cum: 6-coumarin; CMF: cell membrane fragments; IL: Intralipid^®^ 10%. Figure S6: Flow cytometry internalization of 6-cum labelled IL in F98 cells, with different IL/cell ratio. Right panel: iMFI; ratio vs. ctrl (untreated cells), central panel: iMFI; left panel: iMFI/cell number. Abbreviations: 6-cum: 6-coumarin; IL: Intralipid^®^ 10%; iMFI: integrated mean fluorescence intensity. Conditions: (1) 1 μL IL vs. 1 × 10^5^ cells; (2) 1 μL IL vs. 5 × 10^5^ cells; (3) 1 μL IL vs. 1 × 10^6^ cells; (4) 1 μL IL vs. 5 × 10^6^ cells; (5) 1 μL IL vs. 1 × 10^7^ cells. Figure S7: Irinotecan lactone/hydroxyacid transition, SN38 conversion and internalization within 2D-cultured F98 cells. Upper panel: scheme of irinotecan lactone/hydroxyacid transition and SN38 conversion (adapted from: Li, W.Y., Koda, R.T. Stability of irinotecan hydrochloride in aqueous solutions. *Am J Health Syst Pharm.* **2002**, *59(6)*, 539–544. doi:10.1093/ajhp/59.6.539); lower right panel: cell medium; lower left panel: cells. Abbreviations: AOT: sodium docusate; CMF: cell membrane fragments; IL: Intralipid^®^ 10%; SN38: 7-ethyl-10-hidroxy-campthotecin. Statistical analysis: * *p* < 0.05 vs. irinotecan; ** *p* < 0.01 vs. irinotecan; # *p* < 0.05 vs. irinotecan–AOT loaded IL. Figure S8: Apoptosis in F98 cells treated with F98 CMF-wrapped IL (F98 CMF IL), free doxorubicin (dox), doxorubicin–AOT-loaded IL (dox-AOT IL), and doxorubicin–AOT-loaded F98 CMF-wrapped IL (dox-AOT F98 CMF IL). (**A**) The flow cytometry profiles of a representative experiment in annexin V/PI-stained cells at 24 h are shown. Q1-LL = live (annexin V−/PI−), Q1-UL = necrosis (annexin V−/PI+), Q1-LR = early stage of apoptosis (annexin V+/PI−), Q1-UR = late stage of apoptosis (annexin V+/PI+), and (**B**) histograms reporting cytofluorimetric analysis of annexin V/PI staining in F98 treated sublines. Early and late apoptosis were expressed as means ± SEM of three independent experiments. Abbreviations: AOT: sodium socusate; CMF: cell membrane fragments; dox: doxorubicin; IL: Intralipid^®^ 10%; propidium iodide (PI). Statistical analysis: ## *p* < 0.01 vs. F98 CMF IL-treated cells. Figure S9: Transwell experiments with hCMEC/D3 monolayer. (**A**) FITC-dextran permeation across the hCMEC/D3 monolayer. (B) Doxorubicin in the apical chamber over time. (**C**) Doxorubicin flow into the basolateral chamber over time. Abbreviations: AOT: sodium docusate; CMF: cell membrane fragments; FITC: fluorescein isothiocianate; hCMEC/D3: human cerebral microvascular endothelial cells; IL: Intralipid^®^ 10%. Statistical analysis: * *p* < 0.05 vs. doxorubicin. Figure S10: MTT assay at 24 and 48 h, in F98 cells, of doxorubicin–AOT-loaded, unfunctionalized and F98 CMF-wrapped IL. Left panel: 24 h; right panel: 48 h. Abbreviations: AOT: sodium docusate; IL: Intralipid^®^ 10%; CMF: cell membrane fragments; MTT: 3-(4,5-dimethylthiazol-2-yl)-2,5-diphenyltetrazolium bromide. Statistical analysis: * *p* < 0.05 vs. doxorubicin. Figure S11: MTT assay at 72 h, in F98 cells, of doxorubicin–AOT-loaded, unfunctionalized and RBC CMF-wrapped IL. Abbreviations: AOT: sodium docusate; IL: Intralipid^®^ 10%; CMF: cell membrane fragments; MTT: 3-(4,5-dimethylthiazol-2-yl)-2,5-diphenyltetrazolium bromide; RBC: red blood cells.

## References

[1] Ferraris C., Cavalli R., Panciani P.P., Battaglia L. (2020). Overcoming the Blood-Brain Barrier: Successes and Challenges in Developing Nanoparticle-Mediated Drug Delivery Systems for the Treatment of Brain Tumours. Int. J. Nanomed..

[2] Arko L., Katsyv I., Park G.E., Luan W.P., Park J.K. (2010). Experimental approaches for the treatment of malignant gliomas. Pharmacol. Ther..

[3] Tapeinos C., Battaglini M., Ciofani G. (2017). Advances in the design of solid lipid nanoparticles and nanostructured lipid carriers for targeting brain diseases. J. Control Release.

[4] Koukourakis M.I., Koukourakis S., Fezoulidis I., Kelekis N., Kyrias G., Archimandritis S., Karkavitsas N. (2000). High intratumoural accumulation of stealth liposomal doxorubicin (Caelyx) in glioblastomas and in metastatic brain tumours. Br. J. Cancer.

[5] Hau P., Dietrich J., Fabel K., Bogdahn U. (2002). Advances in the therapy of high-grade glioma at relapse: Pegylated liposomal doxorubicin. Expert. Rev. Neurother..

[6] Elinzano H., Toms S., Robison J., Mohler A., Carcieri A., Cielo D., Donnelly J., Disano D., Vatketich J., Baekey J. (2021). Nanoliposomal Irinotecan and Metronomic Temozolomide for Patients With Recurrent Glioblastoma: BrUOG329, A Phase I Brown University Oncology Research Group Trial. Am. J. Clin. Oncol..

[7] Hippalgaonkar K., Majumdar S., Kansara V. (2010). Injectable lipid emulsions-advancements, opportunities and challenges. AAPS PharmSciTech..

[8] Battaglia L.S., Dorati R., Maestrelli F., Conti B., Gabriele M., Di Cesare Mannelli L., Selmin F., Cosco D. (2022). Repurposing of parenterally administered active substances used to treat pain both systemically and locally. Drug Discov. Today.

[9] Hu C.M., Zhang L., Aryal S., Cheung C., Fang R.H., Zhang L. (2011). Erythrocyte membrane-camouflaged polymeric nanoparticles as a biomimetic delivery platform. Proc. Natl. Acad. Sci. USA.

[10] Copp J.A., Fang R.H., Luk B.T., Hu C.M., Gao W., Zhang K., Zhang L. (2014). Clearance of pathological antibodies using biomimetic nanoparticles. Proc. Natl. Acad. Sci. USA.

[11] Fan Z., Zhou H., Li P.Y., Speer J.E., Cheng H. (2014). Structural elucidation of cell membrane-derived nanoparticles using molecular probes. J. Mater. Chem. B..

[12] Cao H., Dan Z., He X., Zhang Z., Yu H., Yin Q., Li Y. (2016). Liposomes Coated with Isolated Macrophage Membrane Can Target Lung Metastasis of Breast Cancer. ACS Nano.

[13] Harris J.C., Scully M.A., Day E.S. (2019). Cancer Cell Membrane-Coated Nanoparticles for Cancer Management. Cancers.

[14] Li H., Jin K., Luo M., Wang X., Zhu X., Liu X., Jiang T., Zhang Q., Wang S., Pang Z. (2019). Size Dependency of Circulation and Biodistribution of Biomimetic Nanoparticles: Red Blood Cell Membrane-Coated Nanoparticles. Cells.

[15] Ben-Akiva E., Meyer R.A., Yu H., Smith J.T., Pardoll D.M., Green J.J. (2020). Biomimetic anisotropic polymeric nanoparticles coated with red blood cell membranes for enhanced circulation and toxin removal. Sci. Adv..

[16] Yaman S., Chintapula U., Rodriguez E., Ramachandramoorthy H., Nguyen K.T. (2020). Cell-mediated and cell membrane-coated nanoparticles for drug delivery and cancer therapy. Cancer Drug Resist..

[17] Foglietta F., Bozza A., Ferraris C., Cangemi L., Bordano V., Serpe L., Martina K., Lazzarato L., Pizzimenti S., Grattarola M. (2023). Surface Functionalised Parenteral Nanoemulsions for Active and Homotypic Targeting to Melanoma. Pharmaceutics.

[18] Battaglia L., Muntoni E., Chirio D., Peira E., Annovazzi L., Schiffer D., Mellai M., Riganti C., Salaroglio I.C., Lanotte M. (2017). Solid lipid nanoparticles by coacervation loaded with a methotrexate prodrug: Preliminary study for glioma treatment. Nanomedicine.

[19] Biasibetti E., Valazza A., Capucchio M.T., Annovazzi L., Battaglia L., Chirio D., Gallarate M., Mellai M., Muntoni E., Peira E. (2017). Comparison of Allogeneic and Syngeneic Rat Glioma Models by Using MRI and Histopathologic Evaluation. Comp. Med..

[20] Spötl L., Sarti A., Dierich M.P., Möst J. (1995). Cell membrane labeling with fluorescent dyes for the demonstration of cytokine-induced fusion between monocytes and tumor cells. Cytometry.

[21] Battaglia L., Gallarate M., Peira E., Chirio D., Muntoni E., Biasibetti E., Capucchio M.T., Valazza A., Panciani P.P., Lanotte M. (2014). Solid lipid nanoparticles for potential doxorubicin delivery in glioblastoma treatment: Preliminary in vitro studies. J. Pharm. Sci..

[22] Poudel B.K., Gupta B., Ramasamy T., Thapa R.K., Youn Y.S., Choi H.G., Yong C.S., Kim J.O. (2016). Development of polymeric irinotecan nanoparticles using a novel lactone preservation strategy. Int. J. Pharm..

[23] Feng L., De Dille A., Jameson V.J., Smith L., Dernell W.S., Manning M.C. (2004). Improved potency of cisplatin by hydrophobic ion pairing. Cancer Chemother. Pharmacol..

[24] Li W.Y., Koda R.T. (2002). Stability of irinotecan hydrochloride in aqueous solutions. Am. J. Health Syst. Pharm..

[25] Raghavan R., Burchett M., Loffredo D., Mulligan J.A. (2000). Low-level (PPB) determination of cisplatin in cleaning validation (rinse water) samples. II. A high-performance liquid chromatographic method. Drug Dev. Ind. Pharm..

[26] Gallarate M., Trotta M., Battaglia L., Chirio D. (2010). Cisplatin-loaded SLN produced by coacervation technique. J. Drug Del. Sci. Technol..

[27] Dianzani C., Monge C., Miglio G., Serpe L., Martina K., Cangemi L., Ferraris C., Mioletti S., Osella S., Gigliotti C.L. (2020). Nanoemulsions as Delivery Systems for Poly-Chemotherapy Aiming at Melanoma Treatment. Cancers.

[28] Monge C., Stoppa I., Ferraris C., Bozza A., Battaglia L., Cangemi L., Miglio G., Pizzimenti S., Clemente N., Gigliotti C.L. (2022). Parenteral Nanoemulsions Loaded with Combined Immuno- and Chemo-Therapy for Melanoma Treatment. Nanomaterials.

[29] Ristroph K.D., Prud’homme R.K. (2019). Hydrophobic ion pairing: Encapsulating small molecules, peptides, and proteins into nanocarriers. Nanoscale Adv..

[30] Howng S.L., Wu C.H., Cheng T.S., Sy W.D., Lin P.C., Wang C., Hong Y.R. (2002). Differential expression of Wnt genes, beta-catenin and E-cadherin in human brain tumors. Cancer Lett..

[31] Edwards L.A., Woolard K., Son M.J., Li A., Lee J., Ene C., Mantey S.A., Maric D., Song H., Belova G. (2011). Effect of brain- and tumor-derived connective tissue growth factor on glioma invasion. J. Natl. Cancer Inst..

[32] Gao X., Xia X., Li F., Zhang M., Zhou H., Wu X., Zhong J., Zhao Z., Zhao K., Liu D. (2021). Circular RNA-encoded oncogenic E-cadherin variant promotes glioblastoma tumorigenicity through activation of EGFR-STAT3 signalling. Nat. Cell Biol..

[33] Troyanovsky R.B., Sokolov E.P., Troyanovsky S.M. (2006). Endocytosis of cadherin from intracellular junctions is the driving force for cadherin adhesive dimer disassembly. Mol. Biol. Cell..

[34] Troyanovsky S. (2005). Cadherin dimers in cell-cell adhesion. Eur. J. Cell Biol..

[35] Rosenberg B., VanCamp L., Trosko J.E., Mansour V.H. (1969). Platinum compounds: A new class of potent antitumour agents. Nature..

[36] Fujiwara Y., Tatsumi M. (1977). Cross-link repair in human cells and its possible defect in Fanconi’s anemia cells. J. Mol. Biol..

[37] Rice J.A., Crothers D.M., Pinto A.L., Lippard S.J. (1988). The major adduct of the antitumor drug cis-diamminedichloroplatinum(II) with DNA bends the duplex by approximately equal to 40 degrees toward the major groove. Proc. Natl. Acad. Sci. USA.

[38] Fischer S.J., Benson L.M., Fauq A., Naylor S., Windebank A.J. (2008). Cisplatin and dimethyl sulfoxide react to form an adducted compound with reduced cytotoxicity and neurotoxicity. Neurotoxicology.

[39] Kobayashi K., Bouscarel B., Matsuzaki Y., Ceryak S., Kudoh S., Fromm H. (1999). pH-dependent uptake of irinotecan and its active metabolite, SN-38, by intestinal cells. Int. J. Cancer..

[40] Cummings J., Boyd G., Macpherson J.S., Wolf H., Smith G., Smyth J.F., Jodrell D.I. (2002). Factors influencing the cellular accumulation of SN-38 and camptothecin. Cancer Chemother. Pharmacol..

[41] Liu X., Hummon A.B. (2015). Quantitative determination of irinotecan and the metabolite SN-38 by nanoflow liquid chromatography-tandem mass spectrometry in different regions of multicellular tumor spheroids. J. Am. Soc. Mass. Spectrom..

[42] Najlah M., Kadam A., Wan K.W., Ahmed W., Taylor K.M.G., Elhissi A.M.A. (2016). Novel paclitaxel formulations solubilized by parenteral nutrition nanoemulsions for application against glioma cell lines. Int. J. Pharm..

[43] De Pasquale D., Pucci C., Desii A., Marino A., Debellis D., Leoncino L., Prato M., Moscato S., Amadio S., Fiaschi P. (2023). A Novel Patient-Personalized Nanovector Based on Homotypic Recognition and Magnetic Hyperthermia for an Efficient Treatment of Glioblastoma Multiforme. Adv. Healthc. Mater..

[44] Yano T., Nakayama R., Ushijima K. (2000). Intracerebroventricular propofol is neuroprotective against transient global ischemia in rats: Extracellular glutamate level is not a major determinant. Brain Res..

[45] Gragasin F.S., Davidge S.T., Tsui B.C.H. (2009). The potential use of intralipid to minimize propofol’s cardiovascular effects. Can. J. Anesth..

[46] Weinberg G.L. (2012). Lipid emulsion infusion: Resuscitation for local anesthetic and other drug overdose. Anesthesiology.

[47] Liu Y., Zhang J., Yu P., Niu J., Yu S. (2021). Mechanisms and Efficacy of Intravenous Lipid Emulsion Treatment for Systemic Toxicity From Local Anesthetics. Front. Med..

[48] Di Pietro S., Falcone A., Arfuso F., Pennisi M., Piccione G., Giudice E. (2022). Treatment of Permethrin Toxicosis in Cats by Intravenous Lipid Emulsion. Toxics.

[49] Anez-Bustillos L., Dao D.T., Baker M.A., Fell G.L., Puder M., Gura K.M. (2016). Intravenous Fat Emulsion Formulations for the Adult and Pediatric Patient: Understanding the Differences. Nutr. Clin. Pract. Off. Publ. Am. Soc. Parenter. Enter. Nutr..

[50] Hanson A.J., Banks W.A., Bettcher L.F., Pepin R., Raftery D., Craft S. (2020). Cerebrospinal fluid lipidomics: Effects of an intravenous triglyceride infusion and apoE status. Metabolomics.

[51] Eldor J. (2017). Intralipid treatment for Alzheimer Disease. Clin. Med. Investig..

[52] Muntoni E., Marini E., Ferraris C., Garelli S., Capucchio M.T., Colombino E., Panciani P.P., Battaglia L. (2022). Intranasal lipid nanocarriers: Uptake studies with fluorescently labeled formulations. Colloids Surf. B. Biointerfaces.

[53] Groothuis D.R. (2000). The blood-brain and blood-tumor barriers: A review of strategies for increasing drug delivery. Neuro Oncol..

[54] Liu Y., Lu W. (2012). Recent advances in brain tumor-targeted nano-drug delivery systems. Expert. Opin. Drug Deliv..

[55] Hoarau-Véchot J., Rafii A., Touboul C., Pasquier J. (2018). Halfway between 2D and Animal Models: Are 3D Cultures the Ideal Tool to Study Cancer-Microenvironment Interactions?. Int. J. Mol. Sci..

[56] Longati P., Jia X., Eimer J., Wagman A., Witt M.R., Rehnmark S., Verbeke C., Toftgård R., Löhr M., Heuchel R.L. (2013). 3D pancreatic carcinoma spheroids induce a matrix-rich, chemoresistant phenotype offering a better model for drug testing. BMC Cancer.

[57] Cavo M., Fato M., Peñuela L., Beltrame F., Raiteri R., Scaglione S. (2016). Microenvironment complexity and matrix stiffness regulate breast cancer cell activity in a 3D in vitro model. Sci. Rep..

[58] Vitale C., Marzagalli M., Scaglione S., Dondero A., Bottino C., Castriconi R. (2022). Tumor Microenvironment and Hydrogel-Based 3D Cancer Models for In Vitro Testing Immunotherapies. Cancers.

[59] Dai X., Ma C., Lan Q., Xu T. (2016). 3D bioprinted glioma stem cells for brain tumor model and applications of drug susceptibility. Biofabrication.

[60] Khurana A., Godugu C., Rehm B., Moradali M. Alginate-Based Three-Dimensional In Vitro Tumor Models: A Better Alternative to Current Two-Dimensional Cell Culture Models. Alginates and Their Biomedical Applications.

[61] Marrella A., Dondero A., Aiello M., Casu B., Olive D., Regis S., Bottino C., Pende D., Meazza R., Caluori G. (2019). Cell-Laden Hydrogel as a Clinical-Relevant 3D Model for Analyzing Neuroblastoma Growth, Immunophenotype, and Susceptibility to Therapies. Front. Immunol..

[62] Pelizzoni G., Scaglione S. (2023). 3D Human Tumor Tissues Cultured in Dynamic Conditions as Alternative In Vitro Disease Models. Methods Mol. Biol..

[63] Trujillo-de Santiago G., Flores-Garza B.G., Tavares-Negrete J.A., Lara-Mayorga I.M., González-Gamboa I., Zhang Y.S., Rojas-Martínez A., Ortiz-López R., Álvarez M.M. (2019). The Tumor-on-Chip: Recent Advances in the Development of Microfluidic Systems to Recapitulate the Physiology of Solid Tumors. Materials.

[64] Marrella A., Varani G., Aiello M., Vaccari I., Vitale C., Mojzisek M., Degrassi C., Scaglione S. (2021). 3D fluid-dynamic ovarian cancer model resembling systemic drug administration for efficacy assay. ALTEX.

[65] Marzagalli M., Pelizzoni G., Fedi A., Vitale C., Fontana F., Bruno S., Poggi A., Dondero A., Aiello M., Castriconi R. (2022). A multi-organ-on-chip to recapitulate the infiltration and the cytotoxic activity of circulating NK cells in 3D matrix-based tumor model. Front. Bioeng. Biotechnol..

