# Supplementary material for: Cell Membrane Fragment-Wrapped Parenteral Nanoemulsions: A New Drug Delivery Tool to Target Gliomas
Source: Cells. 2024 Apr 6;13(7):641. doi: 10.3390/cells13070641 (PMC11011487; doi:10.3390/cells13070641)
Supplement: Supplementary file 1 [file cells-13-00641-s001.zip › cells-2769066-supplementary.pdf]

Cell Membrane Fragment-Wrapped Parenteral Nanoemulsions: A New Drug Delivery Tool to Target Gliomas

Chiara Dianzani, Annalisa Bozza, Valentina Bordano, Luigi Cangemi, Chiara Ferraris, Federica Foglietta, Chiara Monge, Margherita Gallicchio, Stefania Pizzimenti, Elisabetta Marini, Elisabetta Muntoni, Maria Carmen Valsania, Luigi Battaglia

Supplementary materials

Figure S1 Page 2

Figure S2 Page 3

Table S1 Page 4

Figure S3 Page 5

Figure S4 Page 6

Figure S5 Page 7

Figure S6 Page 8

Figure S7 Page 9

Figure S8 Page 10

Figure S9 Page 11

Figure S10 Page 12

Figure S11 Page 13

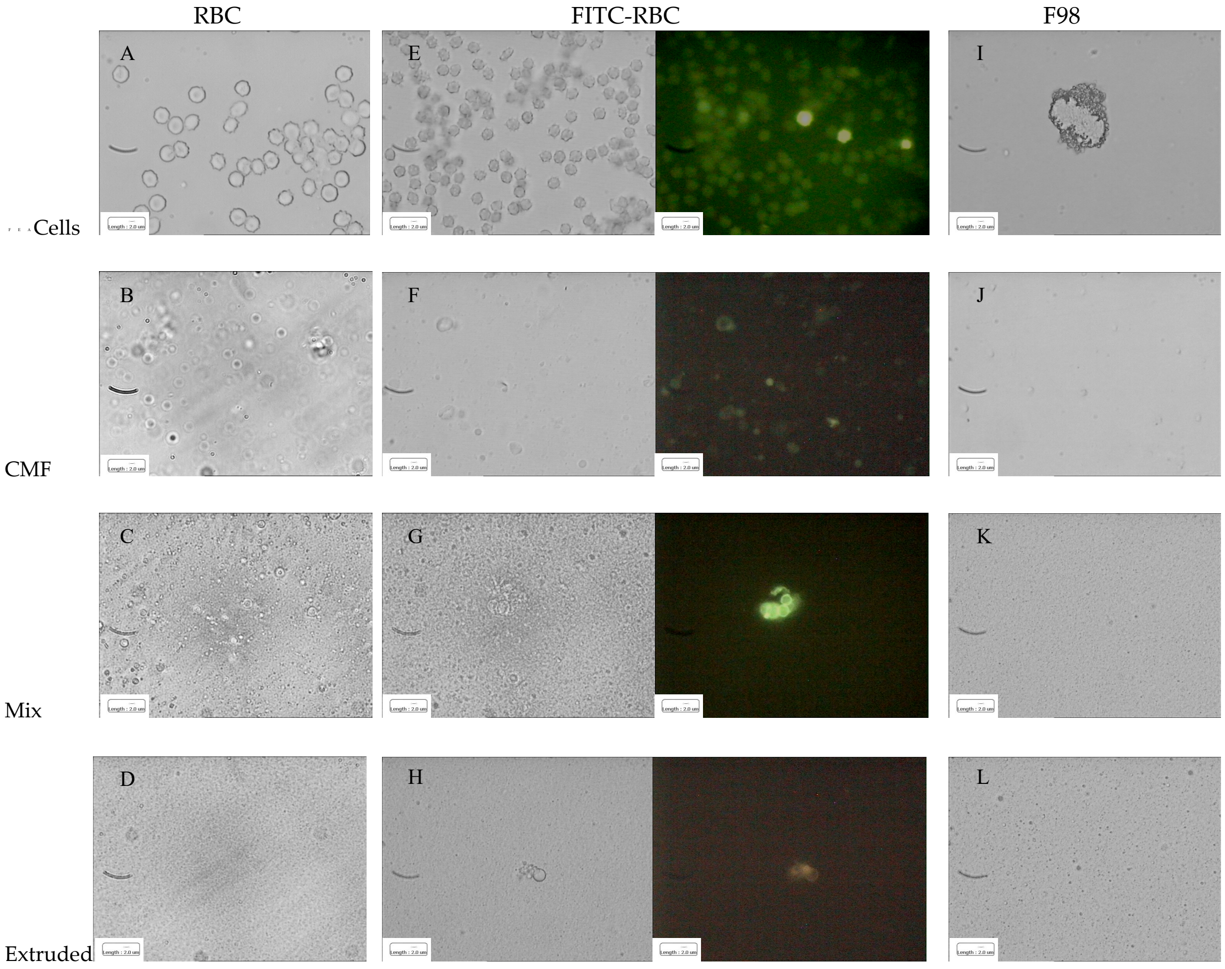

Figure S1. Optical microscopy of IL wrapping process with RBC and F98 CMF. A-D: RBC cells, CMF, CMF + IL mix, CMF/IL mix after extrusion and resuspension. E-H: FITC–RBC cells, CMF, CMF + IL mix, CMF/IL mix after extrusion and resuspension. I-L: F98 cells, CMF, CMF + IL mix, CMF/IL mix after extrusion and resuspension. Paired fluorescence and normal-light images are reported for FITC-labelled RBC. Abbreviations: CMF: cell membrane fragments; FITC: fluorescein isothiocyanate; IL: Intralipid® 10%; RBC: red blood cells.

A) F98 CMF

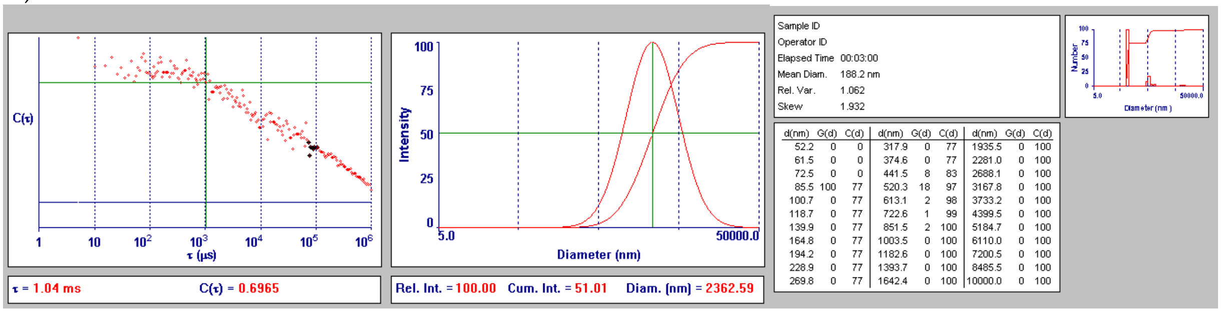

B) RBC CMF

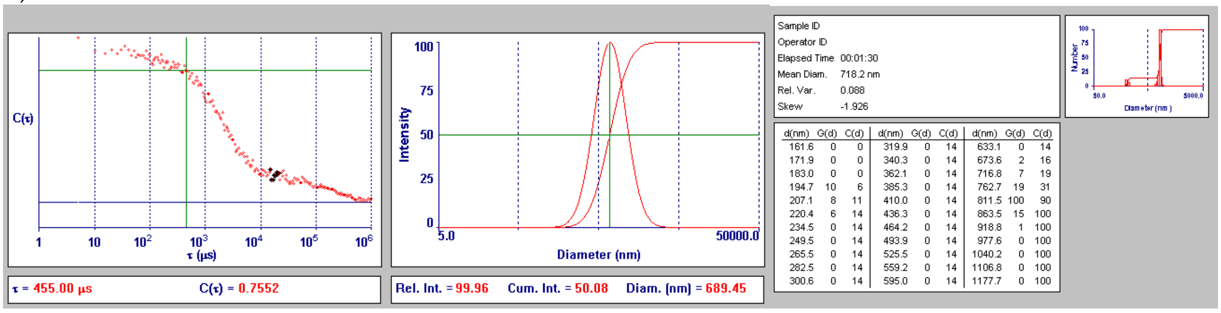

Figure S2. Raw DLS data of F98 (A) and RBC (B) CMF: autocorrelation curve; lognomal size distribution; MSD summary by number of particles. For MSD lecithin was hypothesized as the main component of CMF, with a refractive index=1.459 (Bansal, N.; Truong, T.; Bhandari, B. Feasibility study of lecithin nanovesicles as spacers to improve the solubility of milk protein concentrate powder during storage. *Dairy Sci. Technol.* **2017**, *96*, 861–872, doi:10.1007/s13594-016-0307-0). Abbreviations: CMF: cell membrane fragments; DLS: dynamic light scattering; MSD: multimodal size distribution; RBC: red blood cells.

Table S1. Ion pair characterization. Percentage purity was calculated as the percentage ratio between measured and theoretical drug mg_{eq} for each mg ion pair. Abbreviations: AOT: sodium docusate; mg_{eq}: mg equivalent.

Drug	Ion pair	Molar ratio	mg_{eq} drug / 1 mg ion pair		% purity
			Theoretical	Measured	
Doxorubicin hydrochloride	Doxorubicin–AOT	1:1	0.61	0.56	91.8
Irinotecan hydrochloride	Irinotecan–AOT	1:1	0.64	0.57	89.1
Cisplatin	Cisplatin–AOT	1:2	0.28	0.068	24.3

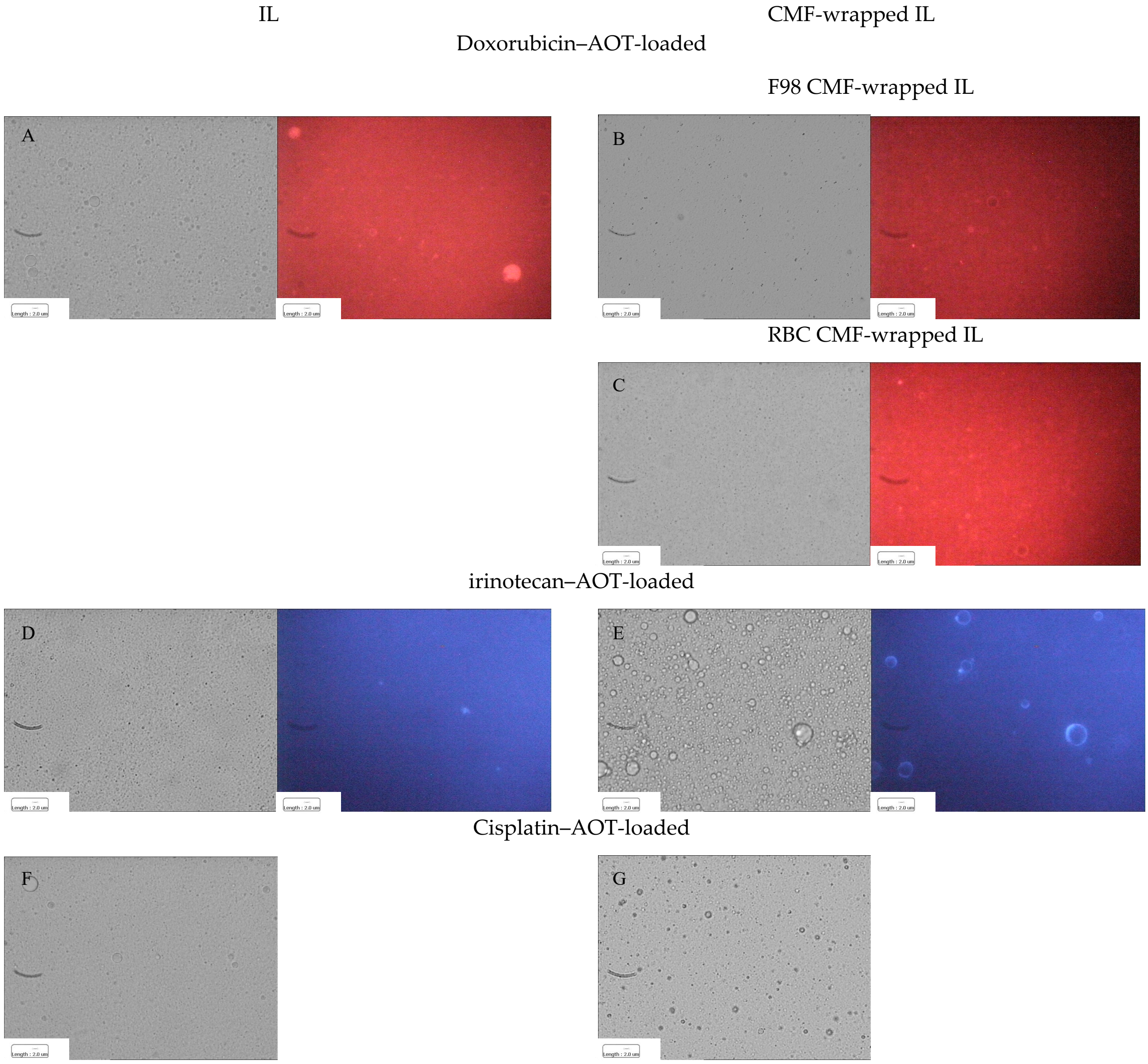

Figure S3. Optical microscopy (florescence and normal light) of drug (doxorubicin–AOT, irinotecan–AOT, cisplatin–AOT) loaded, unfunctionalized and RBC and F98 CMF-wrapped nanoemulsions. A-C: doxorubicin–AOT loaded IL: unfunctionalized, F98 CMF wrapped, RBC CMF wrapped; D-E: irinotecan–AOT-loaded IL: unfunctionalized, F98 CMF-wrapped; F-G: cisplatin–AOT-loaded IL: unfunctionalized, F98 CMF-wrapped. Paired fluorescence and normal-light images are reported for doxorubicin–AOT and irinotecan–AOT-loaded formulations. Abbreviations: AOT: sodium docusate; CMF: cell membrane fragments; RBC: red blood cells.

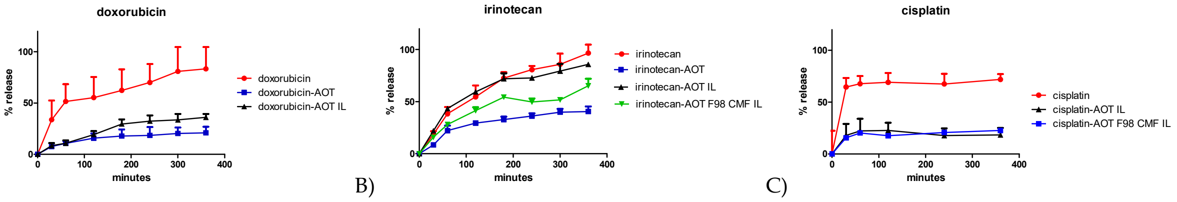

A) B) C)

Figure S4. Drug (doxorubicin, irinotecan, cisplatin) release from doxorubicin–AOT, irinotecan–AOT, cisplatin–AOT loaded nanoemulsions. A: Doxorubicin; B: irinotecan; C: cisplatin. Abbreviations: AOT: sodium docusate; CMF: cell membrane fragments.

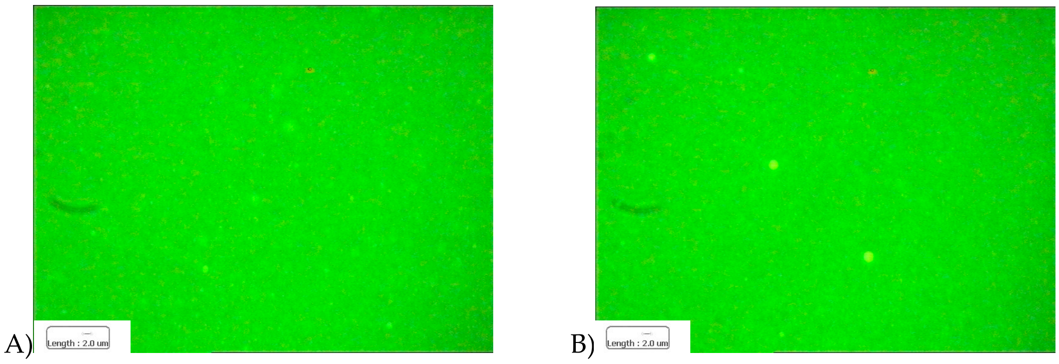

Figure S5. Fluorescence microscopy of 6-cum labelled nanoemulsions. A: IL; B: F98 CMF-wrapped IL. 630x magnification. Abbreviations: 6-cum: 6-coumarin; CMF: cell membrane fragments; IL: Intralipid® 10%.

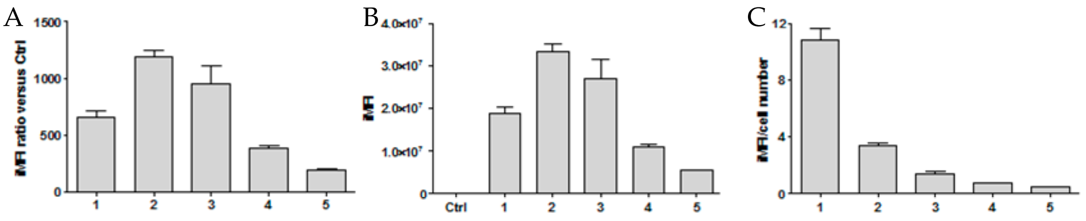

Figure S6. Flow cytometry internalization of 6-cum labelled IL in F98 cells, with different IL/cell ratio. A: iMFI ratio vs ctrl (untreated cells); B: iMFI; C: iMFI/cell number. Abbreviations: 6-coumarin; IL: Intralipid® 10%; iMFI: integrated mean fluorescence intensity. Conditions: 1) 1 μL IL *vs* 1 x 10^5 cells ; 2) 1 μL IL *vs* 5 x 10^5 cells; 3) 1 μL IL *vs* 1 x 10^6 cells; 4) 1 μL IL *vs* 5 x 10^6 cells; 5) 1 μL IL *vs* 1 x 10^7 cells.

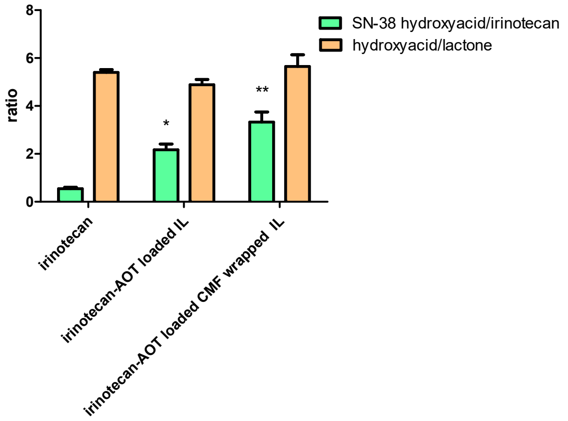

A)

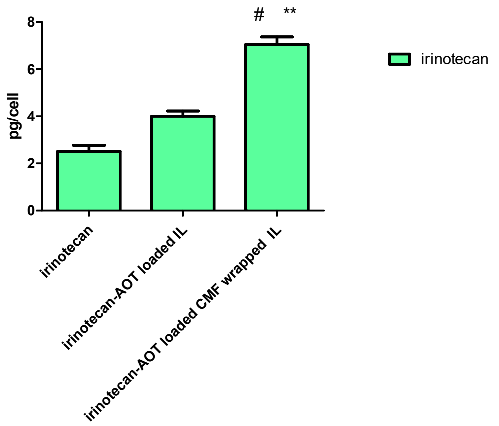

B)

Figure S7. Irinotecan lactone/hydroxyacid transition, SN38 conversion and internalization within 2D-cultured F98 cells. A: Cell medium; B: cells. Abbreviations: AOT: sodium docusate; CMF: cell membrane fragments; IL: Intralipid® 10%; SN38: 7-ethyl-10-hidroxy-campthotecin. Statistical analysis: * p<0.05 *vs* irinotecan; ** p<0.01 *vs* irinotecan; # p<0.05 vs irinotecan-AOT loaded IL.

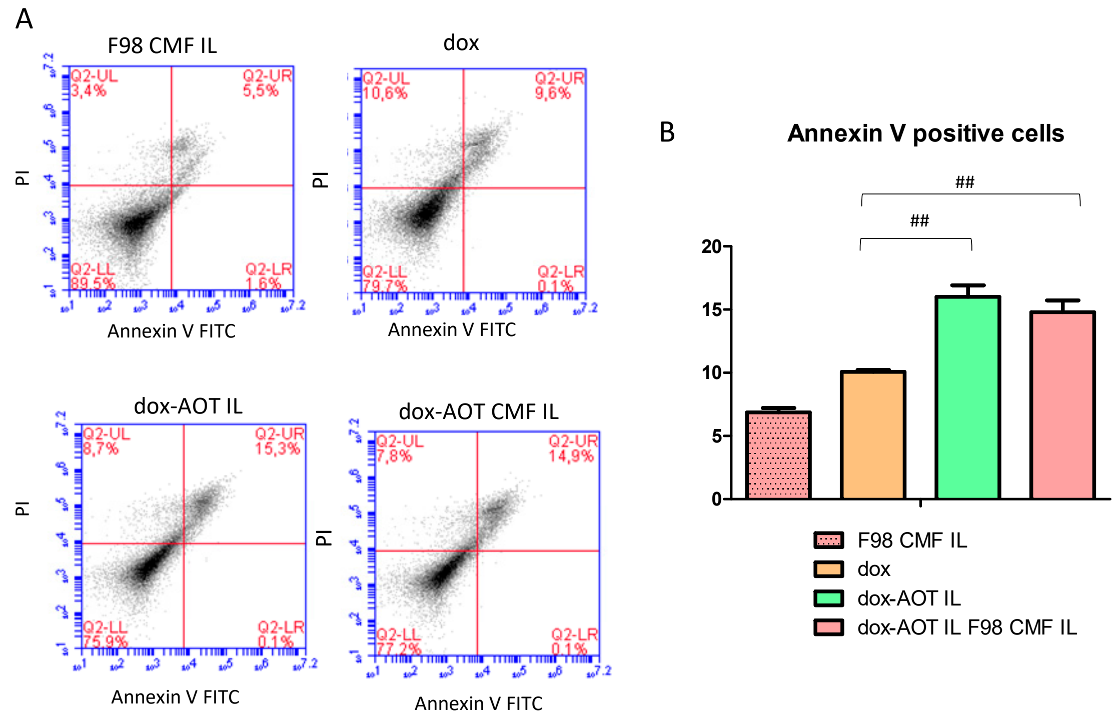

Figure S8. Apoptosis in F98 cells treated with F98 CMF-wrapped IL (F98 CMF IL), free doxorubicin (dox), doxorubicin–AOT-loaded IL (dox-AOT IL), and doxorubicin–AOT-loaded F98 CMF-wrapped IL (dox-AOT F98 CMF IL). (A) The flow cytometry profiles of a representative experiment in Annexin V/PI-stained cells at 24 hours are shown. Q1-LL = live (annexin V−/PI−), Q1-UL = necrosis (annexin V−/PI+), Q1-LR = early stage of apoptosis (annexin V+/PI−), Q1-UR = late stage of apoptosis (annexin V+/PI+), and (B) histograms reporting cytofluorimetric analysis of annexin V/PI staining in F98 treated sublines. Early and late apoptosis were expressed as means ± SEM of three independent experiments. Abbreviations: AOT: sodium socusate; CMF: cell membrane fragments; dox: doxorubicin; IL: Intralipid® 10%; propidium iodide (PI). Statistical analysis: ## $p < 0.01$ *vs* F98 CMF IL treated cells.

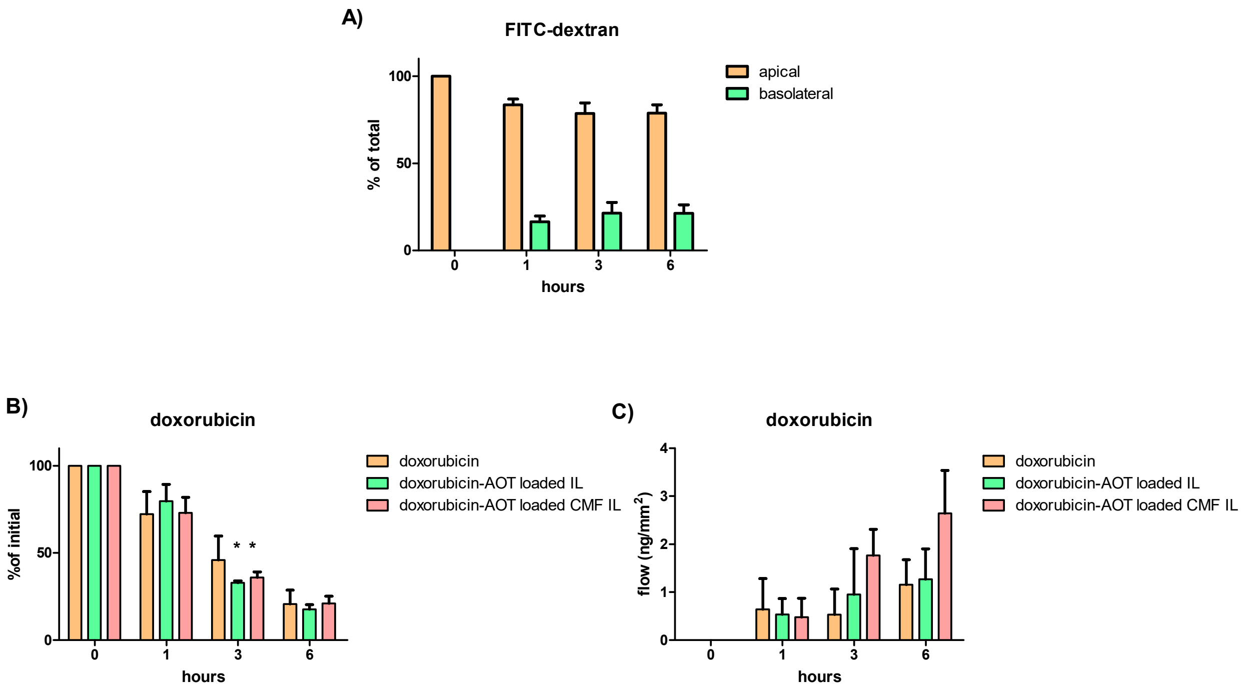

Figure S9. Transwell experiments with hCMEC/D3 monolayer. A) FITC-dextran permeation across the hCMEC/D3 monolayer. B) Doxorubicin in the apical chamber over time. C) Doxorubicin flow into the basolateral chamber over time. Abbreviations: AOT: sodium docusate; CMF: cell membrane fragments; FITC: fluorescein isothiocianate; hCMEC/D3: human cerebral microvascular endothelial cells; IL: Intralipid® 10%. Statistical analysis: * $p<0.05$ vs doxorubicin.

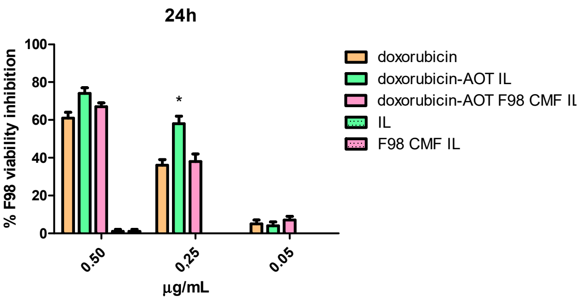

A)

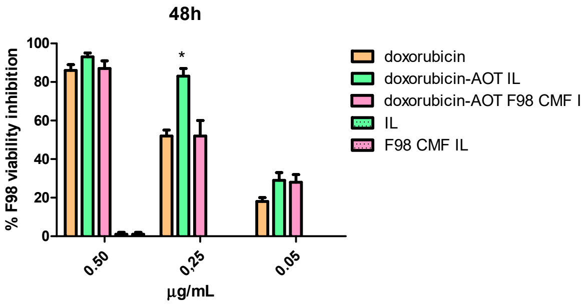

B)

Figure S10. MTT assay at 24 and 48 hours, in F98 cells, of doxorubicin-AOT loaded, unfunctionalized and F98 CMF-wrapped IL. A: 24 hours; B: 48 hours. Abbreviations: AOT: sodium docusate; IL: CMF: cell membrane fragments; Intralipid® 10%; MTT: 3-(4,5-dimethylthiazol-2-yl)-2,5-diphenyltetrazolium bromide. Statistical analysis: * $p<0.05$ *vs* doxorubicin.

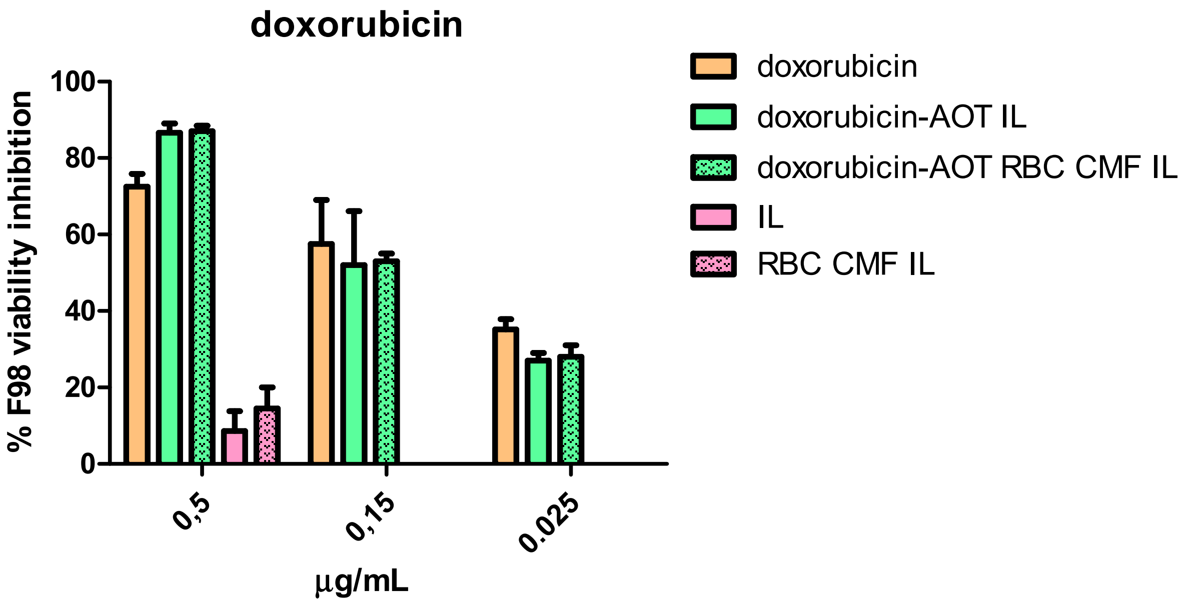

Figure S11. MTT assay at 72 hours, in F98 cells, of doxorubicin-AOT loaded, unfunctionalized and RBC CMF-wrapped IL. Abbreviations: AOT: sodium docusate; IL: CMF: cell membrane fragments; Intralipid® 10%; MTT: 3-(4,5-dimethylthiazol-2-yl)-2,5-diphenyltetrazolium bromide; RBC: red blood cells.
